# Supplementary material for: Granular porous landslide tsunami modelling – the 2014 Lake Askja flank collapse
Source: Nat Commun. 2022 Feb 3;13:678. doi: 10.1038/s41467-022-28296-7 (PMC8813955; doi:10.1038/s41467-022-28296-7)
Supplement: Supplementary file 1 — Supplementary information [file 41467_2022_28296_MOESM1_ESM.pdf]

Supplementary information to ”Granular porous landslide tsunami modelling – the 2014 Lake Askja flank collapse”

Rauter et al.

Contents

1 Derivation of the reduced mathematical model from the three-phase Navier-Stokes Equations 2

2 Meshes and geometry 5
2.1 Laboratory scale cases 5
2.2 Lake Askja case 6

3 Quantitative comparison of the experiment and the simulation 10
3.1 Laboratory Experiment 10
3.2 Lake Askja Study 12

4 Mesh refinement study 12
4.1 Laboratory cases 12
4.2 Lake Askja case 15

5 Sensitivity analysis of laboratory scale simulations 20
5.1 Geometry variation 20
5.2 Friction coefficients 28
5.3 Limiting packing densities 30
5.4 Dynamic dilatancy 32
5.5 Permeability 34
5.6 Particle diameter 36
5.7 Grain density 38
5.8 The effect of a subgrid turbulence model 40

6 Approximation of the experiment with a two-dimensional model 43

7 Sensitivity analysis of Lake Askja landslide 47

8 Centre of mass of slide and water phase in the Lake Askja case 50

1 Derivation of the reduced mathematical model from the three-phase Navier-Stokes Equations

The three-phase Navier-Stokes Equations are given as

$$\frac{\partial \phi_\mathrm{a}}{\partial t} + \nabla \cdot (\phi_\mathrm{a}\, \mathbf{u}_\mathrm{a}) = 0, \tag{1}$$

$$\frac{\partial \phi_\mathrm{w}}{\partial t} + \nabla \cdot (\phi_\mathrm{w}\, \mathbf{u}_\mathrm{w}) = 0, \tag{2}$$

$$\frac{\partial \phi_\mathrm{g}}{\partial t} + \nabla \cdot (\phi_\mathrm{g}\, \mathbf{u}_\mathrm{g}) = 0, \tag{3}$$

$$\frac{\partial \phi_\mathrm{a}\, \rho_\mathrm{a}\, \mathbf{u}_\mathrm{a}}{\partial t} + \nabla \cdot (\phi_\mathrm{a}\, \rho_\mathrm{a}\, \mathbf{u}_\mathrm{a} \otimes \mathbf{u}_\mathrm{a}) = -\phi_\mathrm{a}\, \nabla p + \nabla \cdot (\phi_\mathrm{a}\, \mathbf{T}_\mathrm{a}) + \phi_\mathrm{a}\, \rho_\mathrm{a}\, \mathbf{g} + k_\mathrm{ag}\, (\mathbf{u}_\mathrm{g} - \mathbf{u}_\mathrm{a}) + k_\mathrm{aw}\, (\mathbf{u}_\mathrm{w} - \mathbf{u}_\mathrm{a}), \tag{4}$$

$$\frac{\partial \phi_\mathrm{w}\, \rho_\mathrm{w}\, \mathbf{u}_\mathrm{w}}{\partial t} + \nabla \cdot (\phi_\mathrm{w}\, \rho_\mathrm{w}\, \mathbf{u}_\mathrm{w} \otimes \mathbf{u}_\mathrm{w}) = -\phi_\mathrm{w}\, \nabla p + \nabla \cdot (\phi_\mathrm{w}\, \mathbf{T}_\mathrm{w}) + \phi_\mathrm{w}\, \rho_\mathrm{w}\, \mathbf{g} + k_\mathrm{wg}\, (\mathbf{u}_\mathrm{g} - \mathbf{u}_\mathrm{w}) + k_\mathrm{wa}\, (\mathbf{u}_\mathrm{a} - \mathbf{u}_\mathrm{w}), \tag{5}$$

$$\frac{\partial \phi_\mathrm{g}\, \rho_\mathrm{g}\, \mathbf{u}_\mathrm{g}}{\partial t} + \nabla \cdot (\phi_\mathrm{g}\, \rho_\mathrm{g}\, \mathbf{u}_\mathrm{g} \otimes \mathbf{u}_\mathrm{g}) = -\phi_\mathrm{g}\, \nabla p - \nabla p_\mathrm{s} + \nabla \cdot (\phi_\mathrm{g}\, \mathbf{T}_\mathrm{g}) + \phi_\mathrm{g}\, \rho_\mathrm{g}\, \mathbf{g} + k_\mathrm{ga}\, (\mathbf{u}_\mathrm{a} - \mathbf{u}_\mathrm{g}) + k_\mathrm{gw}\, (\mathbf{u}_\mathrm{w} - \mathbf{u}_\mathrm{g}) \tag{6}$$

with shared pressure p, effective pressure p_s volumetric phase fractions ϕ_i ($i = \mathrm{a, w, g}$), phase velocities $\mathbf{u}_i$, phase densities ρ_i, deviatoric phase stress tensors $\mathbf{T}_i$, drag coefficients k_{ij} and gravitational acceleration $\mathbf{g}$. We want to combine air and water into a single phase and enforce a sharp interface with no momentum diffusion.

Combining the fractions of air and water in a single continuous phase $\phi_\mathrm{c} = \phi_\mathrm{a} + \phi_\mathrm{w}$ with the velocity definition (using the volume averaged velocity)

$$\mathbf{u}_\mathrm{c} = \frac{\phi_\mathrm{a}\, \mathbf{u}_\mathrm{a} + \phi_\mathrm{w}\, \mathbf{u}_\mathrm{w}}{\phi_\mathrm{c}} \tag{7}$$

leads to the mass conservation equation of the continuous phase as

$$\frac{\partial}{\partial t}(\phi_\mathrm{a} + \phi_\mathrm{b}) + \nabla \cdot (\phi_\mathrm{a}\, \mathbf{u}_\mathrm{a} + \phi_\mathrm{w}\, \mathbf{u}_\mathrm{w}) = \frac{\partial \phi_\mathrm{c}}{\partial t} + \nabla \cdot (\phi_\mathrm{c}\, \mathbf{u}_\mathrm{c}) = 0, \tag{8}$$

Using the definition of phase components (note that ϕ_c can not be zero due to always present pores)

$$\alpha_\mathrm{a} = \frac{\phi_\mathrm{a}}{\phi_\mathrm{c}}, \tag{9}$$

$$\alpha_\mathrm{w} = \frac{\phi_\mathrm{w}}{\phi_\mathrm{c}} \tag{10}$$

and the definition of the relative velocity

$$\mathbf{u}_\mathrm{aw} = \mathbf{u}_\mathrm{a} - \mathbf{u}_\mathrm{w}, \tag{11}$$

$$\mathbf{u}_\mathrm{wa} = \mathbf{u}_\mathrm{w} - \mathbf{u}_\mathrm{a}, \tag{12}$$

to express the phase velocities as

$$\mathbf{u}_\mathrm{a} = \mathbf{u}_\mathrm{c} + \alpha_\mathrm{w}\, \mathbf{u}_\mathrm{aw}, \tag{13}$$

$$\mathbf{u}_\mathrm{w} = \mathbf{u}_\mathrm{c} + \alpha_\mathrm{a}\, \mathbf{u}_\mathrm{wa}, \tag{14}$$

allows to write the mass conservation equations of air as

$$\frac{\partial \phi_\mathrm{c}\, \alpha_\mathrm{a}}{\partial t} + \nabla \cdot (\phi_\mathrm{c}\, \alpha_\mathrm{a}\, (\mathbf{u}_\mathrm{c} + \alpha_\mathrm{w}\, \mathbf{u}_\mathrm{aw})) =$$

$$\frac{\partial \phi_\mathrm{c}\, \alpha_\mathrm{a}}{\partial t} + \nabla \cdot (\phi_\mathrm{c}\, \alpha_\mathrm{a}\, \mathbf{u}_\mathrm{c}) + \nabla \cdot (\phi_\mathrm{c}\, \alpha_\mathrm{a}\, \alpha_\mathrm{w}\, \mathbf{u}_\mathrm{aw}) = 0, \tag{15}$$

and similarly for water as

$$\frac{\partial \phi_\mathrm{c}\, \alpha_\mathrm{w}}{\partial t} + \nabla \cdot (\phi_\mathrm{c}\, \alpha_\mathrm{w}\, (\mathbf{u}_\mathrm{c} + \alpha_\mathrm{w}\, \mathbf{u}_\mathrm{wa})) =$$

$$\frac{\partial \phi_\mathrm{c}\, \alpha_\mathrm{w}}{\partial t} + \nabla \cdot (\phi_\mathrm{c}\, \alpha_\mathrm{w}\, \mathbf{u}_\mathrm{c}) + \nabla \cdot (\phi_\mathrm{c}\, \alpha_\mathrm{w}\, \alpha_\mathrm{a}\, \mathbf{u}_\mathrm{wa}) = 0. \tag{16}$$

The relative velocities $\mathbf{u}_\mathrm{aw} = -\mathbf{u}_\mathrm{wa}$ are unknown but we can reconstruct them to enforce a sharp interface and a complete separation of water and air. The reconstruction follows as described to be of the same magnitude as the phase velocity and normal to the interface between air and water (counter gradient transport),

$$\mathbf{u}_\mathrm{aw} = -\mathbf{u}_\mathrm{wa} = c_{\alpha,\mathrm{aw}}\, |\mathbf{u}_\mathrm{c}| \frac{\alpha_\mathrm{w}\, \nabla\, \alpha_\mathrm{a} - \alpha_\mathrm{a}\, \nabla\, \alpha_\mathrm{w}}{|\alpha_\mathrm{w}\, \nabla\, \alpha_\mathrm{a} - \alpha_\mathrm{a}\, \nabla\, \alpha_\mathrm{w}|}. \tag{17}$$

Equations (8), (15), (16) and (17) allow a complete reconstruction of the air and water phase if the combined velocity $\mathbf{u}_\mathrm{c}$ is known. The partial differential equation for the combined velocity $\mathbf{u}_\mathrm{c}$ can derived by summing up the momentum conservation equation of water and air. Various simplifications are required and each term will be simplified individually in the following, starting with the temporal derivative (i.e. inertia):

$$\begin{gathered}
\frac{\partial \phi_\mathrm{a}\,\rho_\mathrm{a}\,\mathbf{u}_\mathrm{a}}{\partial t} + \frac{\partial \phi_\mathrm{w}\,\rho_\mathrm{w}\,\mathbf{u}_\mathrm{w}}{\partial t} = \\
\frac{\partial}{\partial t}\left(\phi_\mathrm{a}\,\rho_\mathrm{a}\,\mathbf{u}_\mathrm{a} + \phi_\mathrm{w}\,\rho_\mathrm{w}\,\mathbf{u}_\mathrm{w}\right) = \\
\frac{\partial}{\partial t}\big(\phi_\mathrm{a}\,\rho_\mathrm{a}\,\left(\mathbf{u}_\mathrm{c} + \alpha_\mathrm{w}\,\mathbf{u}_\mathrm{aw}\right) + \phi_\mathrm{w}\,\rho_\mathrm{w}\,\left(\mathbf{u}_\mathrm{c} - \alpha_\mathrm{w}\,\mathbf{u}_\mathrm{aw}\right)\big) = \\
\frac{\partial}{\partial t}\big(\left(\phi_\mathrm{a}\,\rho_\mathrm{a} + \phi_\mathrm{w}\,\rho_\mathrm{w}\right)\,\mathbf{u}_\mathrm{c} + \left(\phi_\mathrm{a}\,\rho_\mathrm{a}\,\alpha_\mathrm{w} - \phi_\mathrm{w}\,\rho_\mathrm{w}\,\alpha_\mathrm{w}\right)\,\mathbf{u}_\mathrm{aw}\big)
\end{gathered} \tag{18}$$

The definition of the continuous density

$$\rho_\mathrm{c} = \alpha_\mathrm{a}\,\rho_\mathrm{a} + \alpha_\mathrm{w}\,\rho_\mathrm{w} = \frac{\phi_\mathrm{a}\,\rho_\mathrm{a} + \phi_\mathrm{w}\,\rho_\mathrm{w}}{\phi_\mathrm{c}} \tag{19}$$

allows to simplify the first term within the temporal derivative to

$$\frac{\partial}{\partial t}\big(\left(\phi_\mathrm{a}\,\rho_\mathrm{a} + \phi_\mathrm{w}\,\rho_\mathrm{w}\right)\,\mathbf{u}_\mathrm{c}\big) = \frac{\partial}{\partial t}\left(\phi_\mathrm{c}\,\rho_\mathrm{c}\,\mathbf{u}_\mathrm{c}\right). \tag{20}$$

The second term can be simplified as follows

$$\frac{\partial}{\partial t}\big(\left(\phi_\mathrm{a}\,\rho_\mathrm{a}\,\alpha_\mathrm{w} - \phi_\mathrm{w}\,\rho_\mathrm{w}\,\alpha_\mathrm{w}\right)\,\mathbf{u}_\mathrm{aw}\big) = \frac{\partial}{\partial t}\left(\frac{\phi_\mathrm{a}\,\phi_\mathrm{w}}{\phi_\mathrm{c}}\left(\rho_\mathrm{a} - \rho_\mathrm{w}\right)\,\mathbf{u}_\mathrm{aw}\right) \approx 0 \tag{21}$$

and it can be neglected if one of the factors, ϕ_a, ϕ_w or $\mathbf{u}_\mathrm{aw}$ is small. We assume that the phases move together and the relative velocity $\mathbf{u}_\mathrm{aw}$ is solely considered to counteract numerical diffusion. Therefore it is save to assume that $\mathbf{u}_\mathrm{aw} \ll \mathbf{u}_\mathrm{c}$ and this term can be neglected. Further, the product $\phi_\mathrm{a}\,\phi_\mathrm{w}$ is zero following the assumption of a sharp interface.

The convective terms can be summed up as follows:

$$\begin{gathered}
\nabla\cdot\left(\phi_\mathrm{a}\,\rho_\mathrm{a}\,\mathbf{u}_\mathrm{a}\otimes\mathbf{u}_\mathrm{a}\right) + \nabla\cdot\left(\phi_\mathrm{w}\,\rho_\mathrm{w}\,\mathbf{u}_\mathrm{w}\otimes\mathbf{u}_\mathrm{w}\right) = \\
\nabla\cdot\left(\phi_\mathrm{a}\,\rho_\mathrm{a}\,\mathbf{u}_\mathrm{a}\otimes\mathbf{u}_\mathrm{a} + \phi_\mathrm{w}\,\rho_\mathrm{w}\,\mathbf{u}_\mathrm{w}\otimes\mathbf{u}_\mathrm{w}\right) = \\
\nabla\cdot\big(\phi_\mathrm{a}\,\rho_\mathrm{a}\,\left(\mathbf{u}_\mathrm{c} + \alpha_\mathrm{w}\,\mathbf{u}_\mathrm{aw}\right)\otimes\left(\mathbf{u}_\mathrm{c} + \alpha_\mathrm{w}\,\mathbf{u}_\mathrm{aw}\right) + \phi_\mathrm{w}\,\rho_\mathrm{w}\,\left(\mathbf{u}_\mathrm{c} - \alpha_\mathrm{a}\,\mathbf{u}_\mathrm{aw}\right)\otimes\left(\mathbf{u}_\mathrm{c} - \alpha_\mathrm{a}\,\mathbf{u}_\mathrm{aw}\right)\big) = \\
\nabla\cdot\big(\phi_\mathrm{a}\,\rho_\mathrm{a}\,\mathbf{u}_\mathrm{c}\otimes\mathbf{u}_\mathrm{c} + 2\,\phi_\mathrm{a}\,\rho_\mathrm{a}\,\mathrm{sym}\left(\mathbf{u}_\mathrm{c}\otimes\alpha_\mathrm{w}\,\mathbf{u}_\mathrm{aw}\right) + \phi_\mathrm{a}\,\rho_\mathrm{a}\,\alpha_\mathrm{w}^2\,\mathbf{u}_\mathrm{aw}\otimes\mathbf{u}_\mathrm{aw} \\
+\phi_\mathrm{w}\,\rho_\mathrm{w}\,\mathbf{u}_\mathrm{c}\otimes\mathbf{u}_\mathrm{c} - 2\,\phi_\mathrm{w}\,\rho_\mathrm{w}\,\mathrm{sym}\left(\mathbf{u}_\mathrm{c}\otimes\alpha_\mathrm{a}\,\mathbf{u}_\mathrm{aw}\right) + \phi_\mathrm{w}\,\rho_\mathrm{w}\,\alpha_\mathrm{a}^2\,\mathbf{u}_\mathrm{aw}\otimes\mathbf{u}_\mathrm{aw}\big) = \\
\nabla\cdot\big(\left(\phi_\mathrm{a}\,\rho_\mathrm{a} + \phi_\mathrm{w}\,\rho_\mathrm{w}\right)\,\mathbf{u}_\mathrm{c}\otimes\mathbf{u}_\mathrm{c} + 2\,\left(\phi_\mathrm{a}\,\rho_\mathrm{a}\,\alpha_\mathrm{w} - \phi_\mathrm{w}\,\rho_\mathrm{w}\,\alpha_\mathrm{a}\right)\,\mathrm{sym}\left(\mathbf{u}_\mathrm{c}\otimes\mathbf{u}_\mathrm{aw}\right) + \\
\left(\phi_\mathrm{a}\,\rho_\mathrm{a}\,\alpha_\mathrm{w}^2 + \phi_\mathrm{w}\,\rho_\mathrm{w}\,\alpha_\mathrm{a}^2\right)\,\mathbf{u}_\mathrm{aw}\otimes\mathbf{u}_\mathrm{aw}\big) = \\
\nabla\cdot\left(\phi_\mathrm{c}\,\rho_\mathrm{c}\,\mathbf{u}_\mathrm{c}\otimes\mathbf{u}_\mathrm{c} + 2\,\frac{\phi_\mathrm{a}\,\phi_\mathrm{w}}{\phi_\mathrm{c}}\,\left(\rho_\mathrm{a} - \rho_\mathrm{w}\right)\,\mathrm{sym}\left(\mathbf{u}_\mathrm{c}\otimes\mathbf{u}_\mathrm{aw}\right) + \frac{\phi_\mathrm{a}\,\phi_\mathrm{w}}{\phi_\mathrm{c}}\,\left(\alpha_\mathrm{w}\,\rho_\mathrm{a} + \alpha_\mathrm{a}\,\rho_\mathrm{w}\right)\,\mathbf{u}_\mathrm{aw}\otimes\mathbf{u}_\mathrm{aw}\right) \approx \\
\nabla\cdot\left(\phi_\mathrm{c}\,\rho_\mathrm{c}\,\mathbf{u}_\mathrm{c}\otimes\mathbf{u}_\mathrm{c}\right),
\end{gathered} \tag{22}$$

The operator sym is defined as

$$\mathrm{sym}\left(\mathbf{A}\right) = \frac{1}{2}\left(\mathbf{A} + \mathbf{A}^T\right). \tag{23}$$

The relative velocities $\mathbf{u}_\mathrm{aw} = -\mathbf{u}_\mathrm{wa}$ and the product $\phi_\mathrm{a}\,\phi_\mathrm{w}$ are neglected again to simplify the system. This is valid because of the same reasons as mentioned before.

Deviatoric stresses can be combined as

$$\nabla\cdot\left(\phi_\mathrm{a}\,\mathbf{T}_\mathrm{a}\right) + \nabla\cdot\left(\phi_\mathrm{w}\,\mathbf{T}_\mathrm{w}\right) = \nabla\cdot\left(\phi_\mathrm{a}\,\mathbf{T}_\mathrm{a} + \phi_\mathrm{w}\,\mathbf{T}_\mathrm{w}\right) = \nabla\cdot\big(\phi_\mathrm{c}\,\left(\alpha_\mathrm{a}\,\mathbf{T}_\mathrm{a} + \alpha_\mathrm{w}\,\mathbf{T}_\mathrm{w}\right)\big) \tag{24}$$

With the shear rates for air

$$\begin{gathered}
\mathbf{S}_\mathrm{a} = \mathrm{sym}\left(\nabla\,\mathbf{u}_\mathrm{a}\right) - \frac{1}{3}\,\nabla\cdot\left(\mathbf{u}_\mathrm{a}\right)\,\mathbf{I} = \mathrm{sym}\big(\nabla\,\left(\mathbf{u}_\mathrm{c} + \alpha_\mathrm{w}\,\mathbf{u}_\mathrm{aw}\right)\big) - \frac{1}{3}\,\nabla\cdot\left(\mathbf{u}_\mathrm{c} + \alpha_\mathrm{w}\,\mathbf{u}_\mathrm{aw}\right)\,\mathbf{I} = \\
\mathrm{sym}\left(\nabla\,\mathbf{u}_\mathrm{c}\right) - \frac{1}{3}\,\nabla\cdot\left(\mathbf{u}_\mathrm{c}\right)\,\mathbf{I} + \mathrm{sym}\big(\nabla\,\left(\alpha_\mathrm{w}\,\mathbf{u}_\mathrm{aw}\right)\big) - \frac{1}{3}\,\nabla\cdot\left(\alpha_\mathrm{w}\,\mathbf{u}_\mathrm{aw}\right)\,\mathbf{I} = \\
\mathbf{S}_\mathrm{c} + \mathrm{sym}\big(\nabla\,\left(\alpha_\mathrm{w}\,\mathbf{u}_\mathrm{aw}\right)\big) - \frac{1}{3}\,\nabla\cdot\left(\alpha_\mathrm{w}\,\mathbf{u}_\mathrm{aw}\right)\,\mathbf{I}
\end{gathered} \tag{25}$$

and water

$$\mathbf{S}_{\mathrm{w}} = \mathbf{S}_{\mathrm{c}} - \operatorname{sym}\left(\nabla\,(\alpha_{\mathrm{a}}\,\mathbf{u}_{\mathrm{aw}})\right) + \frac{1}{3}\,\nabla\cdot(\alpha_{\mathrm{a}}\,\mathbf{u}_{\mathrm{aw}})\,\mathbf{I} \tag{26}$$

and because air and water are Newtonian fluids, $\mathbf{T}_{\mathrm{i}} = \nu_i\,\mathbf{S}_{\mathrm{i}}$ we can write

$$\begin{gathered}\nabla\cdot\left(\phi_{\mathrm{c}}\,(\alpha_{\mathrm{a}}\,\mathbf{T}_{\mathrm{a}} + \alpha_{\mathrm{w}}\,\mathbf{T}_{\mathrm{w}})\right) = \\ \nabla\cdot\left(\phi_{\mathrm{c}}\,\alpha_{\mathrm{a}}\,\nu_{\mathrm{a}}\,\rho_{\mathrm{a}}\left(\mathbf{S}_{\mathrm{c}} + \operatorname{sym}\left(\nabla\,(\alpha_{\mathrm{w}}\,\mathbf{u}_{\mathrm{aw}})\right) - \frac{1}{3}\,\nabla\cdot(\alpha_{\mathrm{w}}\,\mathbf{u}_{\mathrm{aw}})\,\mathbf{I}\right) + \right. \\ \left. \phi_{\mathrm{c}}\,\alpha_{\mathrm{w}}\,\nu_{\mathrm{w}}\,\rho_{\mathrm{w}}\left(\mathbf{S}_{\mathrm{c}} - \operatorname{sym}\left(\nabla\,(\alpha_{\mathrm{a}}\,\mathbf{u}_{\mathrm{aw}})\right) + \frac{1}{3}\,\nabla\cdot(\alpha_{\mathrm{a}}\,\mathbf{u}_{\mathrm{aw}})\,\mathbf{I}\right)\right) \approx \\ \nabla\cdot\left(\phi_{\mathrm{c}}\,(\alpha_{\mathrm{a}}\,\nu_{\mathrm{a}}\,\rho_{\mathrm{a}} + \alpha_{\mathrm{w}}\,\nu_{\mathrm{w}}\,\rho_{\mathrm{w}})\,\mathbf{S}_{\mathrm{c}}\right)\end{gathered} \tag{27}$$

We neglect the terms that contain $\mathbf{u}_{\mathrm{aw}}$ as before. From this equation, it becomes clear that the effective viscosity of the continuous phase has to be defined as

$$\nu_{\mathrm{c}} = \frac{\alpha_{\mathrm{a}}\,\nu_{\mathrm{a}}\,\rho_{\mathrm{a}} + \alpha_{\mathrm{w}}\,\nu_{\mathrm{a}}\,\rho_{\mathrm{w}}}{\alpha_{\mathrm{a}}\,\rho_{\mathrm{a}} + \alpha_{\mathrm{w}}\,\rho_{\mathrm{w}}} \tag{28}$$

so we can express the deviatoric stress tensor in the usual manner as

$$\nabla\cdot\left(\phi_{\mathrm{c}}\,(\alpha_{\mathrm{a}}\,\mathbf{T}_{\mathrm{a}} + \alpha_{\mathrm{w}}\,\mathbf{T}_{\mathrm{w}})\right) \approx \nabla\cdot(\phi_{\mathrm{c}}\,\nu_{\mathrm{c}}\,\rho_{\mathrm{c}}\,\mathbf{S}_{\mathrm{c}})\,. \tag{29}$$

The drag between water and air scales directly with the relative velocity which is neglected:

$$k_{\mathrm{aw}}\,(\mathbf{u}_{\mathrm{w}} - \mathbf{u}_{\mathrm{a}}) = -k_{\mathrm{wa}}\,(\mathbf{u}_{\mathrm{a}} - \mathbf{u}_{\mathrm{w}}) \approx 0 \tag{30}$$

The remaining drag terms are difficult to express in a consistent manner because the drag models are highly non-linear in ϕ_{a} and ϕ_{w}. Therefore, we make the assumption that

$$k_{\mathrm{ga}}\left(\mathbf{u}_{\mathrm{a}} - \mathbf{u}_{\mathrm{g}}\right) + k_{\mathrm{gw}}\left(\mathbf{u}_{\mathrm{w}} - \mathbf{u}_{\mathrm{g}}\right) \approx k_{\mathrm{gc}}\left(\mathbf{u}_{\mathrm{c}} - \mathbf{u}_{\mathrm{g}}\right) = -k_{\mathrm{cg}}\left(\mathbf{u}_{\mathrm{g}} - \mathbf{u}_{\mathrm{c}}\right). \tag{31}$$

This is correct if α_{a} or α_{w} are zero and thus consistent with a sharp interface.

The remaining terms are linear in ϕ_{a} and ϕ_{c} and can be summed up without requirement for simplifications. The final system as reported in the article is obtained:

$$\frac{\partial\phi_{\mathrm{c}}\,\alpha_{\mathrm{a}}}{\partial t} + \nabla\cdot(\phi_{\mathrm{c}}\,\alpha_{\mathrm{a}}\,\mathbf{u}_{\mathrm{c}}) + \nabla\cdot(\phi_{\mathrm{c}}\,\alpha_{\mathrm{a}}\,\alpha_{\mathrm{w}}\,\mathbf{u}_{\mathrm{aw}}) = 0, \tag{32}$$

$$\frac{\partial\phi_{\mathrm{c}}\,\alpha_{\mathrm{w}}}{\partial t} + \nabla\cdot(\phi_{\mathrm{c}}\,\alpha_{\mathrm{w}}\,\mathbf{u}_{\mathrm{c}}) + \nabla\cdot(\phi_{\mathrm{c}}\,\alpha_{\mathrm{w}}\,\alpha_{\mathrm{w}}\,\mathbf{u}_{\mathrm{wa}}) = 0, \tag{33}$$

$$\frac{\partial\phi_{\mathrm{c}}}{\partial t} + \nabla\cdot(\phi_{\mathrm{c}}\,\mathbf{u}_{\mathrm{c}}) = 0, \tag{34}$$

$$\frac{\partial\phi_{\mathrm{g}}}{\partial t} + \nabla\cdot\left(\phi_{\mathrm{g}}\,\mathbf{u}_{\mathrm{g}}\right) = 0, \tag{35}$$

$$\frac{\partial\phi_{\mathrm{c}}\,\rho_{\mathrm{c}}\,\mathbf{u}_{\mathrm{c}}}{\partial t} + \nabla\cdot(\phi_{\mathrm{a}}\,\rho_{\mathrm{c}}\,\mathbf{u}_{\mathrm{c}}\otimes\mathbf{u}_{\mathrm{c}}) = -\phi_{\mathrm{c}}\,\nabla p + \nabla\cdot(\phi_{\mathrm{c}}\,\rho_{\mathrm{c}}\,\nu_{\mathrm{c}}\,\mathbf{S}_{\mathrm{c}}) + \phi_{\mathrm{c}}\,\rho_{\mathrm{c}}\,\mathbf{g} + k_{\mathrm{cg}}\left(\mathbf{u}_{\mathrm{g}} - \mathbf{u}_{\mathrm{c}}\right), \tag{36}$$

$$\frac{\partial\phi_{\mathrm{g}}\,\rho_{\mathrm{g}}\,\mathbf{u}_{\mathrm{g}}}{\partial t} + \nabla\cdot\left(\phi_{\mathrm{g}}\,\rho_{\mathrm{g}}\,\mathbf{u}_{\mathrm{g}}\otimes\mathbf{u}_{\mathrm{g}}\right) = -\phi_{\mathrm{g}}\,\nabla p - \nabla p_{\mathrm{s}} + \nabla\cdot\left(\phi_{\mathrm{g}}\,\rho_{\mathrm{g}}\,\nu_{\mathrm{g}}\,\mathbf{S}_{\mathrm{g}}\right) + \phi_{\mathrm{g}}\,\rho_{\mathrm{g}}\,\mathbf{g} + k_{\mathrm{gc}}\left(\mathbf{u}_{\mathrm{c}} - \mathbf{u}_{\mathrm{g}}\right). \tag{37}$$

Granular viscosity ν_{g}, effective pressure p_{s} and drag coefficients k_{ij} have to be expressed with the respective constitutive models [1].

2 Meshes and geometry

2.1 Laboratory scale cases

The mesh for the laboratory scale simulation is shown in Fig. 1. The impact area is shown magnified in Fig. 2 with background colour following the local bulk density ($\rho = \phi_{\mathrm{a}}\,\rho_{\mathrm{a}} + \phi_{\mathrm{w}}\,\rho_{\mathrm{w}} + \phi_{\mathrm{g}}\,\rho_{\mathrm{g}}$) at $t = 0$.

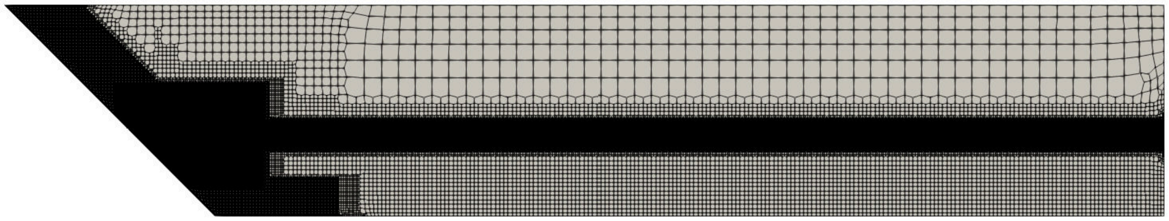

Figure 1: The mesh of the laboratory scale cases. The geometry (spatial simulation domain) is defined by four points. The slide slope, the impact area and the wave region have been refined.

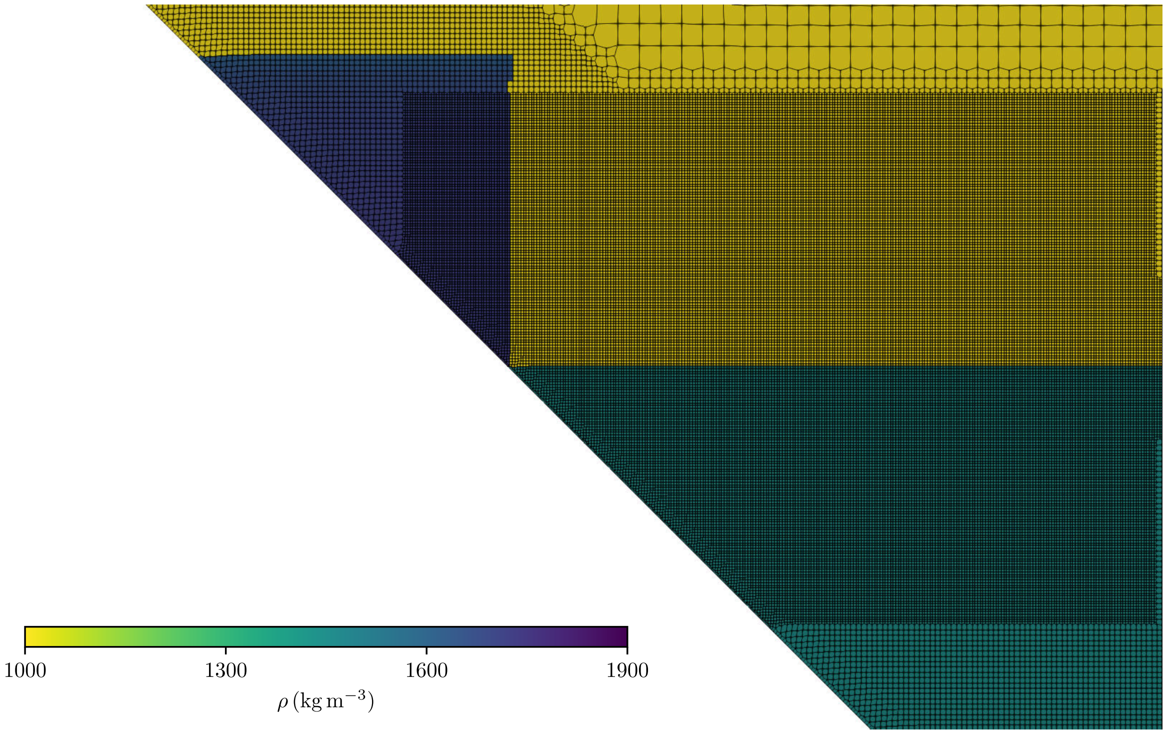

Figure 2: The mesh of the laboratory scale cases. The geometry (spatial simulation domain) is defined by four points. The slide slope, the impact area and the wave region have been refined.

2.2 Lake Askja case

The mesh for the Lake Askja case is based on the terrain as shown in Figs. 3 and 4. First, the geometry is defined by defining the outline of the region of interest and by adding walls and a top lid to the respective region, see Fig. 5. Further, the geometry of the refinement region is defined by a surface as shown in Fig. 6. Cells in the neighbourhood of the water surface (ca. 40 m) will be refined. All data is available in GIS (geographic information system) file formats but the OpenFOAM toolbox is widely based on STL (standard triangulation language) files or similar formats. The terrain data was transferred into STL files with the tools provided by the avalanche module of OpenFOAM [2]. Relevant regions, where a refined mesh is desired, were mapped in GIS and transferred to STL with the same procedure. This includes the free water surface at $z = 1058.25$ m, the path of the landslide and the shores of the lake where inundation is expected. As mentioned in the manuscript, we applied *cartesianMesh* of the cfMesh toolbox [3] to generate a suitable mesh defining the simulation boundary, bottom geometry and local refinements. The final mesh consists of 30.6 M, mostly hexahedral and quadratic cells with cell sizes between 160 m and 2.5 m (corresponding to six refinements levels).

A simplified version of the mesh (cells no smaller than 20 m, they are further refined 3 times for the simulation) is shown in Fig. 7. The lower boundary of the mesh is shown in Fig. 8.

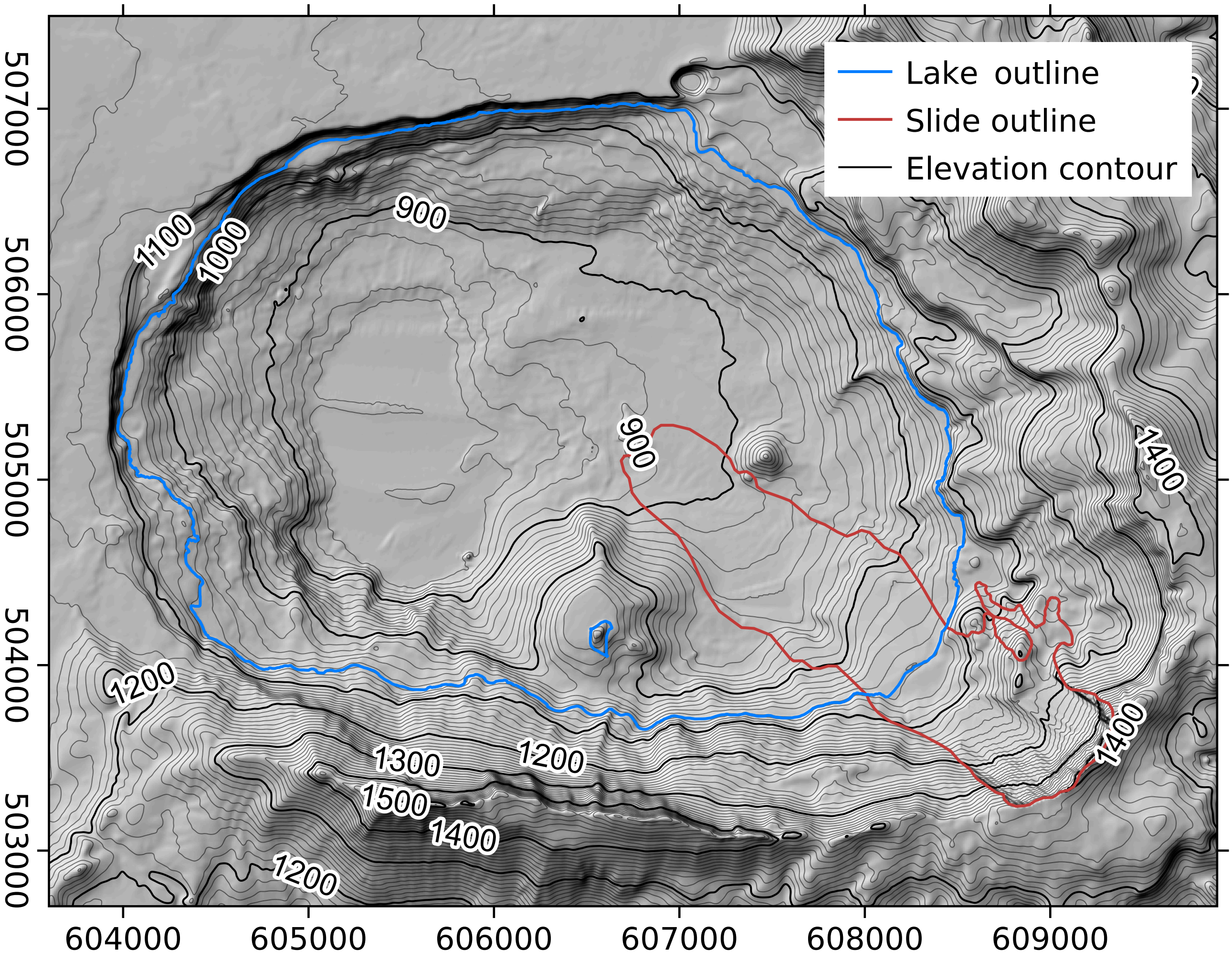

Figure 3: Elevation contour lines of the bathymetry of Lake Askja and its surrounding terrain".

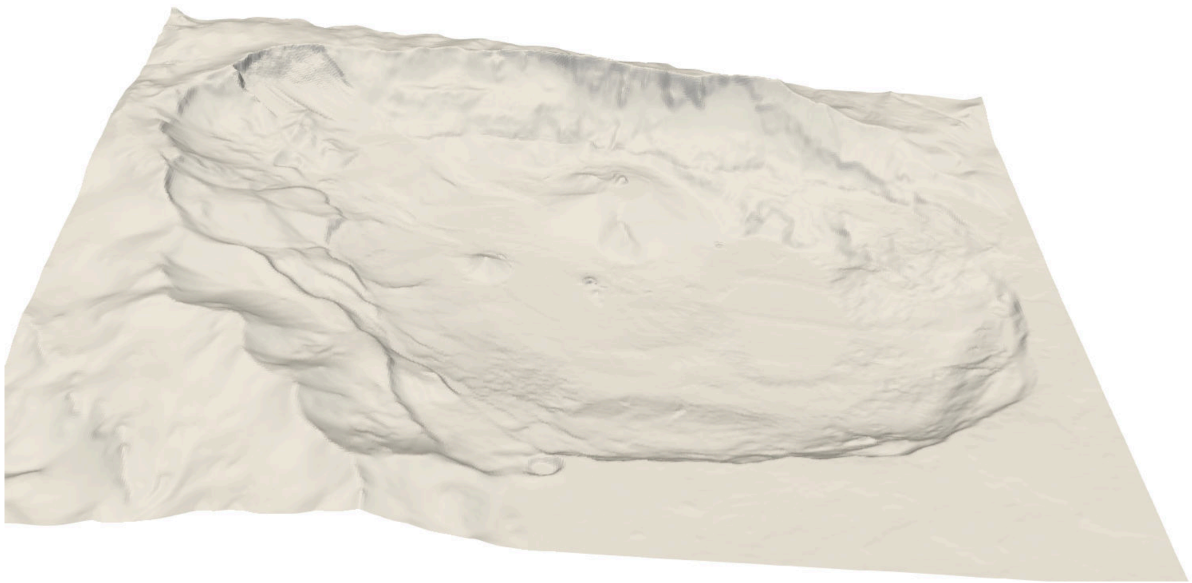

Figure 4: The terrain data without the landslide (neither initial position nor deposition) that was used to generate the mesh for the Lake Askja case.

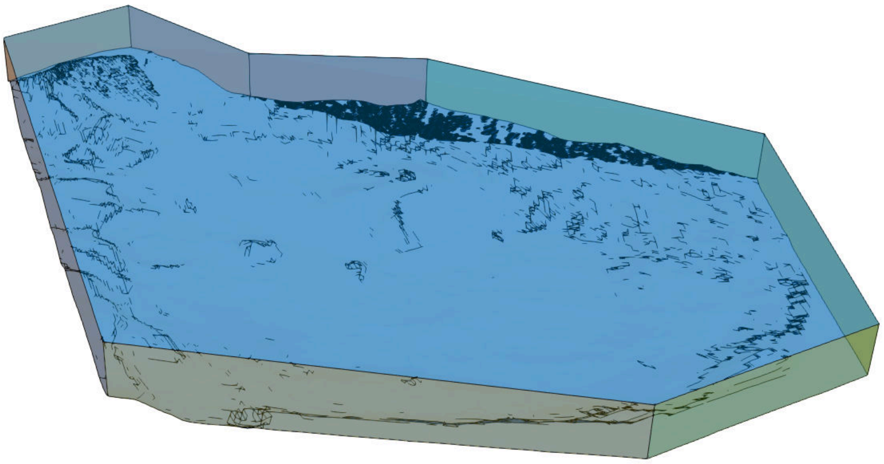

Figure 5: The geometry (Standard Triangulation File) defining the simulation domain. The bottom follows from the terrain model, side walls and top boundary are artificial boundaries.

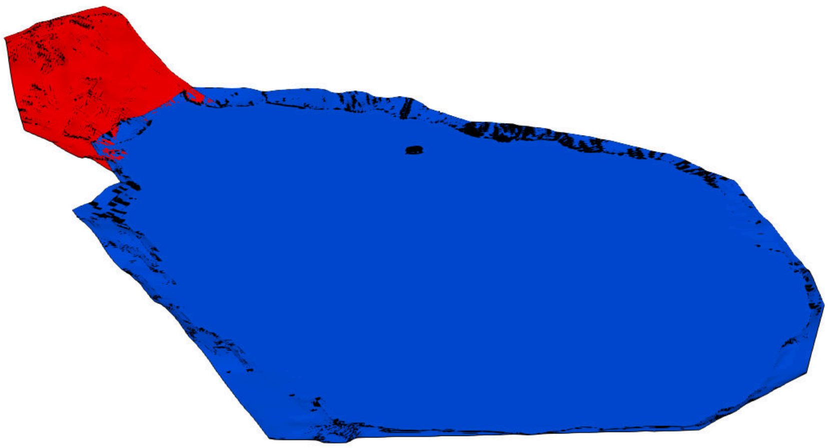

Figure 6: The mesh refinement region was defined by 2D surfaces (Standard Triangulation Files) and the refinement was conducted up to a certain distance (ca. 40 m) from the surface. The red surface shows the refinement region for the landslide and the blue surface shows the refinement region of the lake surface and the inundation region.

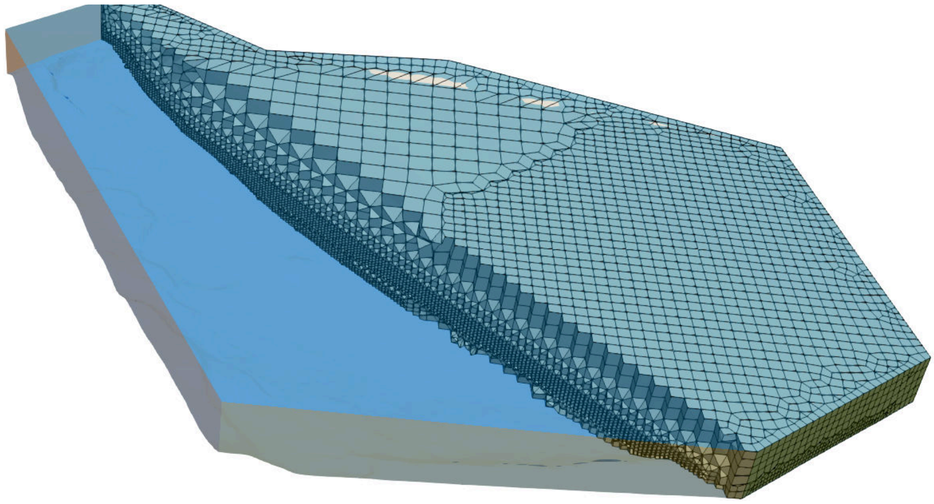

Figure 7: The simulation domain and a part of the final OpenFOAM volume mesh. The complete mesh covers the simulation domain entirely. Note that this is a simplified mesh with cells no smaller than 20 m.

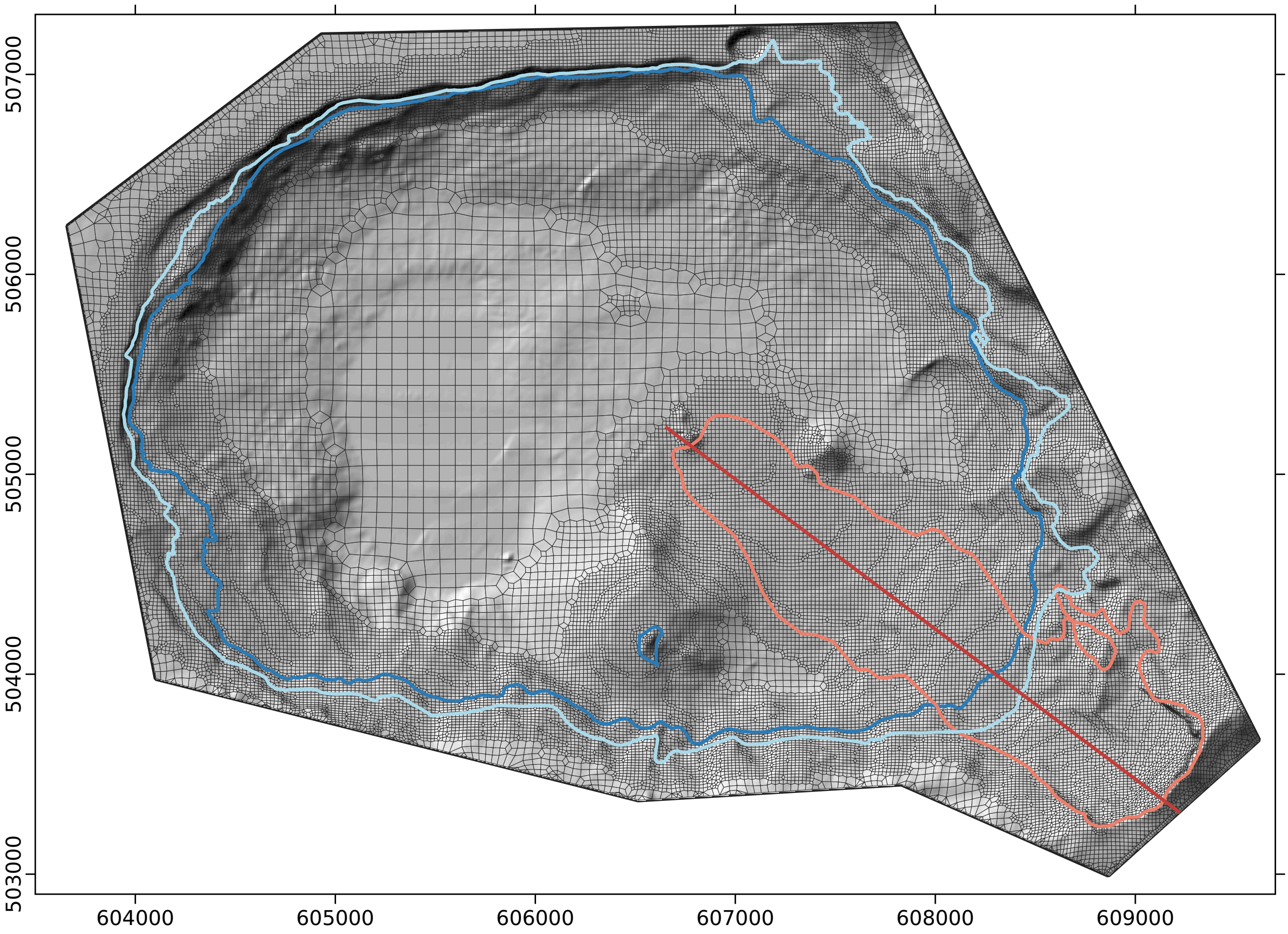

Figure 8: Top view on the lower boundary of the finite volume mesh for the Lake Askja case. The lower boundary represents the terrain surface. The intersection of the lake surface with the terrain (shoreline) is shown in dark blue, the inundation in light blue, the documented affected area of the slide is shown in red. Note that this is a simplified mesh with cells no smaller than 20 m. The refinement covers the slide path, the lake surface and the inundation areas. Note that the middle of the lake appears coarsely meshed because the terrain surface is far away from the free surface, which is refined to the highest level. The coordinates are in the ISN93 geodetic reference system.

3 Quantitative comparison of the experiment and the simulation

3.1 Laboratory Experiment

Multiple quantitative measurements can be introduced to describe the fitness of the simulations to the experiments.

First, there are measurements that take into account the whole time line of a wave signal. A simple quantitative measurement is the L2 error norm of the wave signal at a gauge over the period $[0, T]$, defined as

$$L_2 = \sqrt{\frac{1}{T}\int_0^T (\eta_{\text{sim}} - \eta_{\text{exp}})^2 \,\mathrm{d}t}. \tag{38}$$

Further, we can define the mean error as

$$\Delta\eta_{\text{mean}} = \frac{1}{T}\int_0^T \left|\eta_{\text{sim}} - \eta_{\text{exp}}\right| \,\mathrm{d}t, \tag{39}$$

and the maximum error as

$$\Delta\eta_{\text{max}} = \max_t \left(\left|\eta_{\text{sim}} - \eta_{\text{exp}}\right|\right). \tag{40}$$

The respective results for the example from the manuscript are shown in Tab. 1.

Table 1: Different error definitions for the wave signal at all gauges for the simulation with $\theta = 45°$ and $m_0 = 2\,\text{kg}$.

gauge	L2 norm (mm)	mean error (mm)	maximum error (mm)
1	2.07	1.55	21.08
2	2.43	1.63	17.41
3	2.68	1.87	15.52
4	3.58	2.27	19.52

We can also compare maxima and minima of the wave signal, i.e. crests and troughs. This is also useful from a practical point of view, as the amplitudes are among the most important results, indicating the hazard represented by the wave. Extrema are characterized by arrival time (time of extrema) and amplitude (function value of the extrema). We detect up to two crests and extrema automatically and filter out low peaks (less than 2 mm than the next opposite extrema) and short peaks (less than 0.3 s between extrema). The respective results for the example of the manuscript are shown in Tab. 2. Notably, the errors in arrival time of the second crest and trough at the first wave gauge are very high. This can be traced back to a slightly different wave form at this gauge, introducing an additional crest and trough in the simulation.

Table 2: Quantitative comparison between the experiment and simulation for $\theta = 45^\circ$ and $m_0 = 2\,\mathrm{kg}$.

gauge	crest	a_{exp} (mm)	a_{sim} (mm)	Δa (mm)	$t_{\mathrm{arr,exp}}$ (s)	$t_{\mathrm{arr,sim}}$ (s)	Δt_{arr} (s)
1	1	21.0	21.1	0.082	0.7	0.7	0.000
1	2	2.6	1.1	1.570	1.7	1.2	0.445
2	1	17.4	17.4	0.043	0.9	0.9	0.012
2	2	5.8	5.6	0.171	1.7	1.7	0.072
3	1	15.5	15.5	0.010	1.2	1.2	0.018
3	2	8.6	7.9	0.684	2.1	2.0	0.060
4	1	14.7	14.4	0.261	1.4	1.4	0.024
4	2	19.3	19.5	0.267	2.5	2.4	0.072
gauge	trough	a_{exp} (mm)	a_{sim} (mm)	Δa (mm)	$t_{\mathrm{arr,exp}}$ (s)	$t_{\mathrm{arr,sim}}$ (s)	Δt_{arr} (s)
1	1	-13.5	-11.3	2.164	1.0	1.0	0.024
1	2	-2.2	-1.2	1.051	1.9	1.4	0.547
2	1	-14.6	-12.0	2.564	1.4	1.3	0.024
2	2	-0.2	-2.8	2.589	2.0	1.9	0.078
3	1	-13.9	-11.4	2.474	1.7	1.7	0.030
3	2	0.2	-0.4	0.569	2.4	2.3	0.072
4	1	-11.9	-8.6	3.268	2.0	1.9	0.048

3.2 Lake Askja Study

The L2 error of the inundation height $h_{\max}$ across the periphery of the lake (θ being the azimuth in relation to east) is defined as

$$L_2 = \sqrt{\frac{1}{360^\circ} \int_{0^\circ}^{360^\circ} \left(h_{\max,\text{sim}}(\theta) - h_{\max,\text{field}}(\theta)\right)^2 \, \mathrm{d}\theta}, \tag{41}$$

where $h_{\max,\text{field}}$ is the field measurement of the inundation height after the event. Further, we can define the mean error of the inundation height as

$$\Delta h_{\max,\text{mean}} = \frac{1}{360^\circ} \int_{0^\circ}^{360^\circ} \left|h_{\max,\text{sim}} - h_{\max,\text{field}}\right| \, \mathrm{d}\theta, \tag{42}$$

and the maximum error as

$$\Delta h_{\max,\max} = \max_{\theta} \left(\left| \eta_{\max,\text{sim}}(\theta) - h_{\max,\text{field}}(\theta) \right| \right). \tag{43}$$

The respective results for the example from the manuscript are shown in Tab. 3.

Table 3: Different error definitions for the inundation for the simulations with the multiphase Navier–Stokes equations, Shallow water equations and the Boussinesq equations.

Model	L2 norm (m)	mean error (m)	maximum error (m)
Navier–Stokes	9.99	7.68	32.24
Shallow water [4]	10.75	8.16	39.52
Boussinesq [4]	8.31	6.04	30.90

4 Mesh refinement study

4.1 Laboratory cases

To investigate the numerical uncertainty, four simulations with varying mesh size have been conducted. All meshes are generated such that a cell boundary aligns with the free surface at rest. This makes sure that the initialisation of the free water surface and its behaviour at rest is equally well represented in all cases. A missmatch of the cell boundary with the free surface at rest would have introduced bigger differences.

The finest mesh size, located at the water surface, varied between 0.001 m and 0.008 m (factor of eight), the coarsest mesh size varied less as further coarsening was not achievable with the applied mesh generator, CfMesh. The results are compared in terms of the L2 error norm at the wave gauges as defined in Eq. (38). The resulting wave gauges are shown in Fig. 10. The L2 error norm for the wave signals at gauges are shown in Tab. 4.

Table 4: L2 error norm in wave signals for different mesh resolutions.

Mesh size	$\Delta x = 0.001\,\text{m}$	$\Delta x = 0.002\,\text{m}$	$\Delta x = 0.004\,\text{m}$	$\Delta x = 0.008\,\text{m}$
Gauge 1	2.066 mm	1.895 mm	1.929 mm	2.182 mm
Gauge 2	2.434 mm	2.029 mm	1.924 mm	2.351 mm
Gauge 3	2.678 mm	2.306 mm	2.183 mm	2.628 mm
Gauge 4	3.580 mm	3.186 mm	2.937 mm	3.791 mm

The simulations of the small scale case are very robust in terms of coarse meshes and even the cell size of 0.008 m (about 1/3 of the wave amplitude) is sufficient to yield reasonable results. It was not possible to define coarser girds for the tank geometry and for the initial geometry of the slide and the reservoir. No dramatic numerical wave diffusion can be observed in this example and we have to conclude that the mesh resolution of 0.001 m is more than sufficient for this case. The travel distance and travel duration of the wave to the gauges is short in this example and this might change for further travelling waves (compared to e.g. Rauter et al. [5]). Generally, we can repeat the suggestion of Rauter et al. [5] and Roenby et al. [6] to use 10 cells along the wave amplitude to avoid artificial wave diffusion. Good results on coarser resolutions are possible but we would suggest a mesh refinement study in such a case.

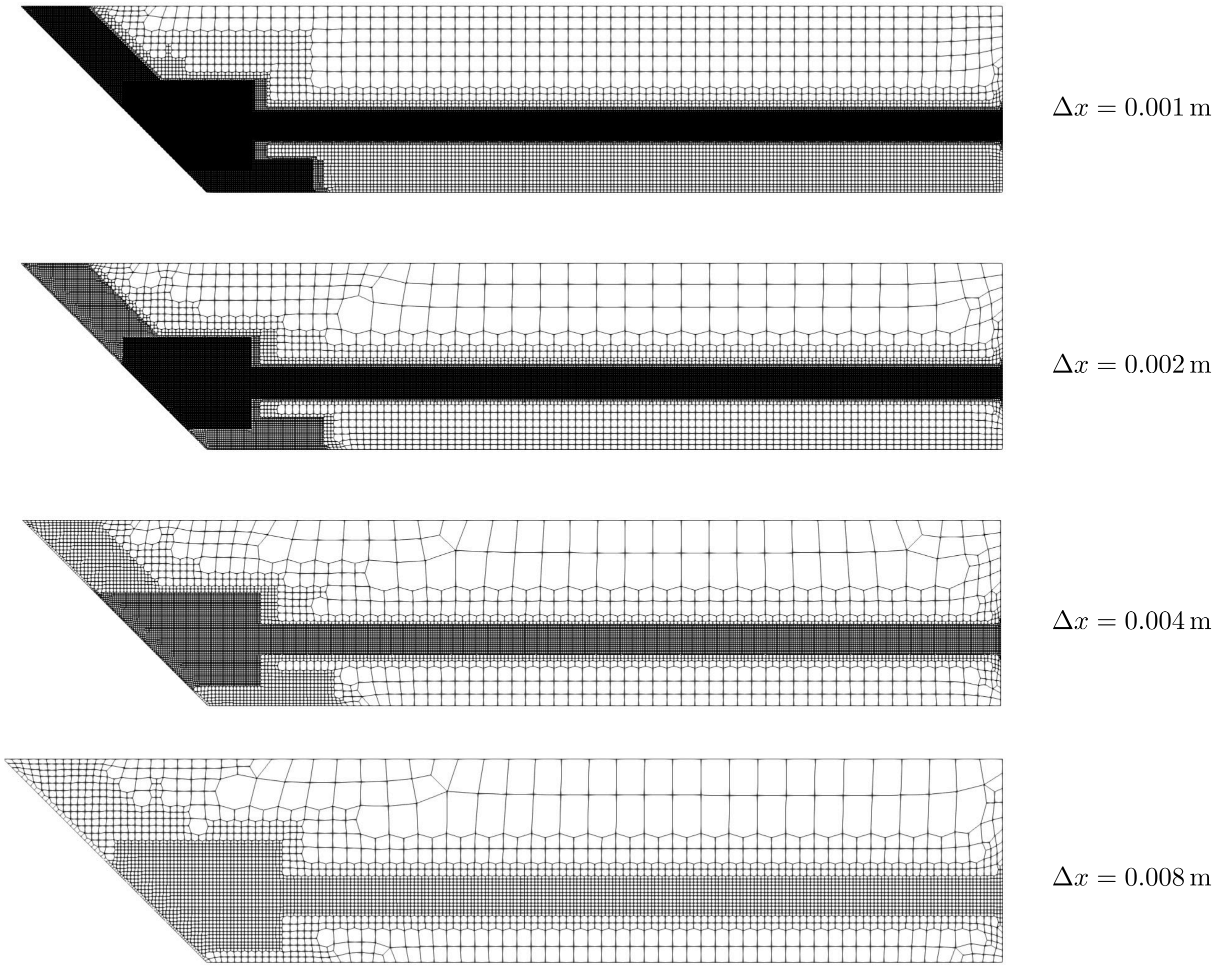

Figure 9: The four meshes used in the mesh refinement study.

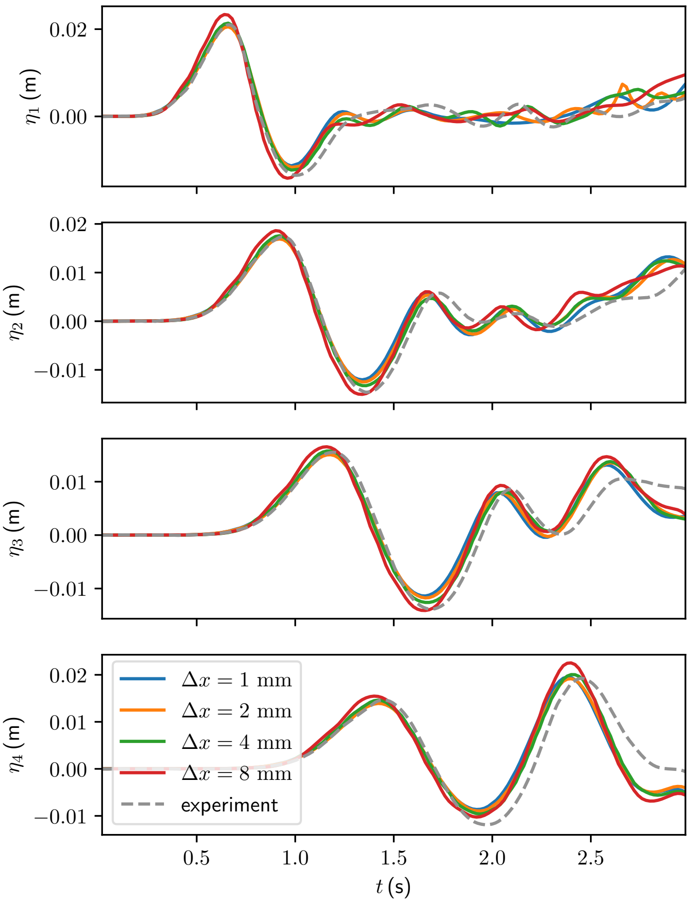

Figure 10: Wave gauges for a variation in the mesh resolution.

4.2 Lake Askja case

For the mesh refinement study of the Lake Askja case, the base mesh with a resolution of 160 m was kept the same. This resolution is required to describe the upper boundary sufficiently accurate to generate a suitable mesh. The refinement steps near the surface were reduced from six (the finest mesh as shown in the manuscript, $\Delta x = 2.5\,\mathrm{m}$) to five ($\Delta x = 5\,\mathrm{m}$) and further to four (coarsest mesh, $\Delta x = 10.0\,\mathrm{m}$). The resulting meshes are shown in Fig. 12. The simulation time is substantially reduced for coarser meshes (see Tab. 5) and there is a non-linear relationship between mesh size, mesh cell numbers and computational time. The reason is that the time step duration has to be reduced for smaller cells, following the CFL conditions (see Rauter [1]) and one would expect that the computational time scales with $T_{\mathrm{execution}} \propto \Delta x^{-5}$, without other limiting factors, such as a maximum time step duration which was 0.004 s in the two coarser simulations and 0.002 s in the finest simulation. Further, the stability is worse for finer meshes and the solver is not scaling perfectly with more CPU cores, that are required to conduct simulations with a high number of mesh cells. The simulations were run for 240 s, as most of the inundation happens before this time.

The simulated slide is shown in Fig. 13 for three different meshes. This sequence shows that the slide behaves similarly in all three simulations with different mesh resolutions. The refined mesh introduces further flow details, but especially in the first stage, which is relevant for the wave generation, these details do not influence the macroscopic kinematics of the slide. However, the bottom boundary layer can not be resolved sufficiently on coarser grids, leading to an underestimation of friction and thus higher velocities and a flow of the full slide into the lake. A similar behaviour was observed by Rauter [1] but it was shown that the model approaches the observed behaviour with increasingly fine meshes. The main error of the simulated slide, that is the overestimation of the northern branch, is similarly present on all meshes. We conclude that this error is not related to numerical uncertainty. From the faster slides it follows that the waves are higher, as visible in Fig. 14 and the same is the case for the inundation, shown in Fig. 11. This leads to an overestimation of the inundation, especially on the northern shore but this is reduced with increasing mesh refinement. The differences in the inundation heights $h_{\max}$ across the periphery of the lake are small and the L2 error norm (with reference to the measured inundation height) are all within 10.4 m and 10.7 m. The high peaks on the inundation height on the northern shore are very pronounced in simulations on a coarse grid but this effect is also reduced with an increasing mesh resolution. This peaks are still overestimated on the finest resolution and it might be that they are further reduced for further mesh refinements.

We conclude from this study that the mesh resolution of 2.5 m is sufficient for the applications and the level of detail in the manuscript. The models behaviour at a higher resolution would be interesting to investigate and we aim to do so in the future, when computational resources are catching up. Further, we can observe that simulations at a lower resolution can still yield useful information and results at a very small computational cost, although the quantitative results should be used with caution.

Table 5: CPU times until $t = 240\,\mathrm{s}$ and errors in the inundation height for three different mesh refinement levels.

Δx	threads	number of cells	CPU time	L2 error	mean error	maximum error
10 m	20	1 221 571	1 425 h	10.7 m	8.1 m	35.4 m
5 m	40	5 044 394	2 850 h	10.4 m	8.1 m	25.9 m
2.5 m	220	30 585 460	139 552 h	10.6 m	8.3 m	32.2 m

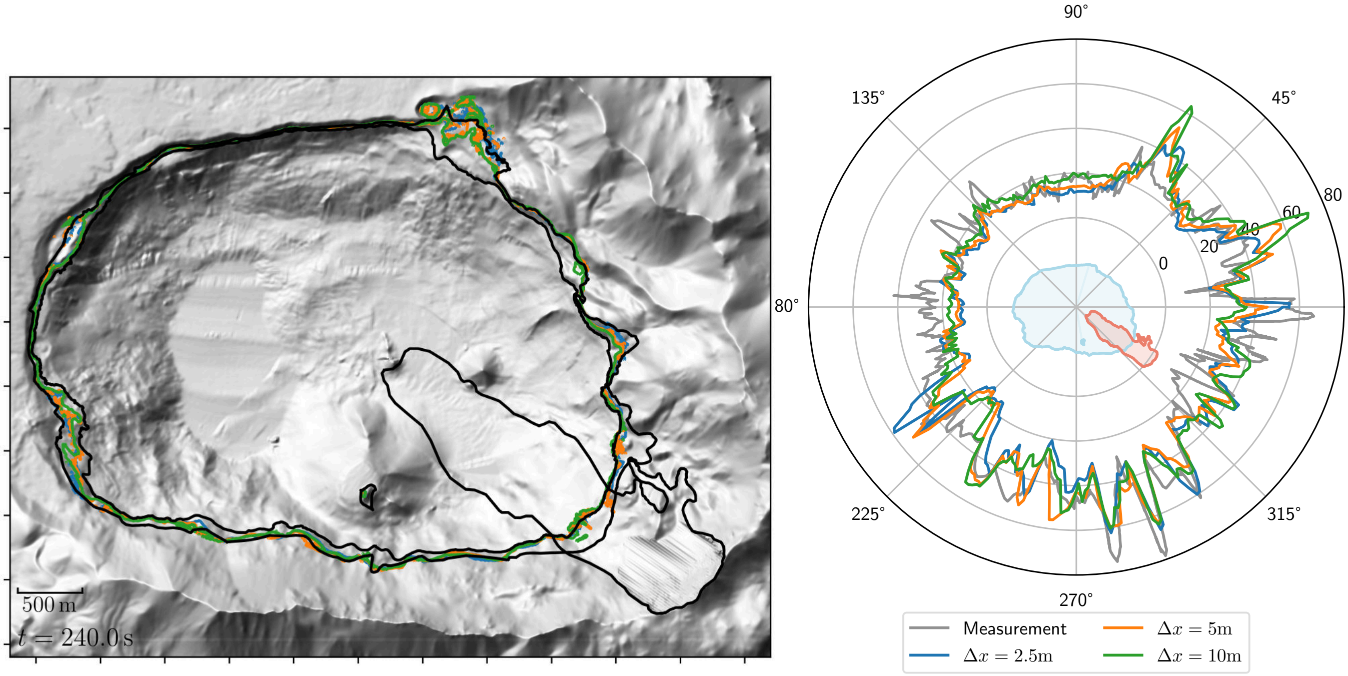

Figure 11: Comparison of the inundation at $t = 240\,\mathrm{s}$ for three different mesh refinement levels.

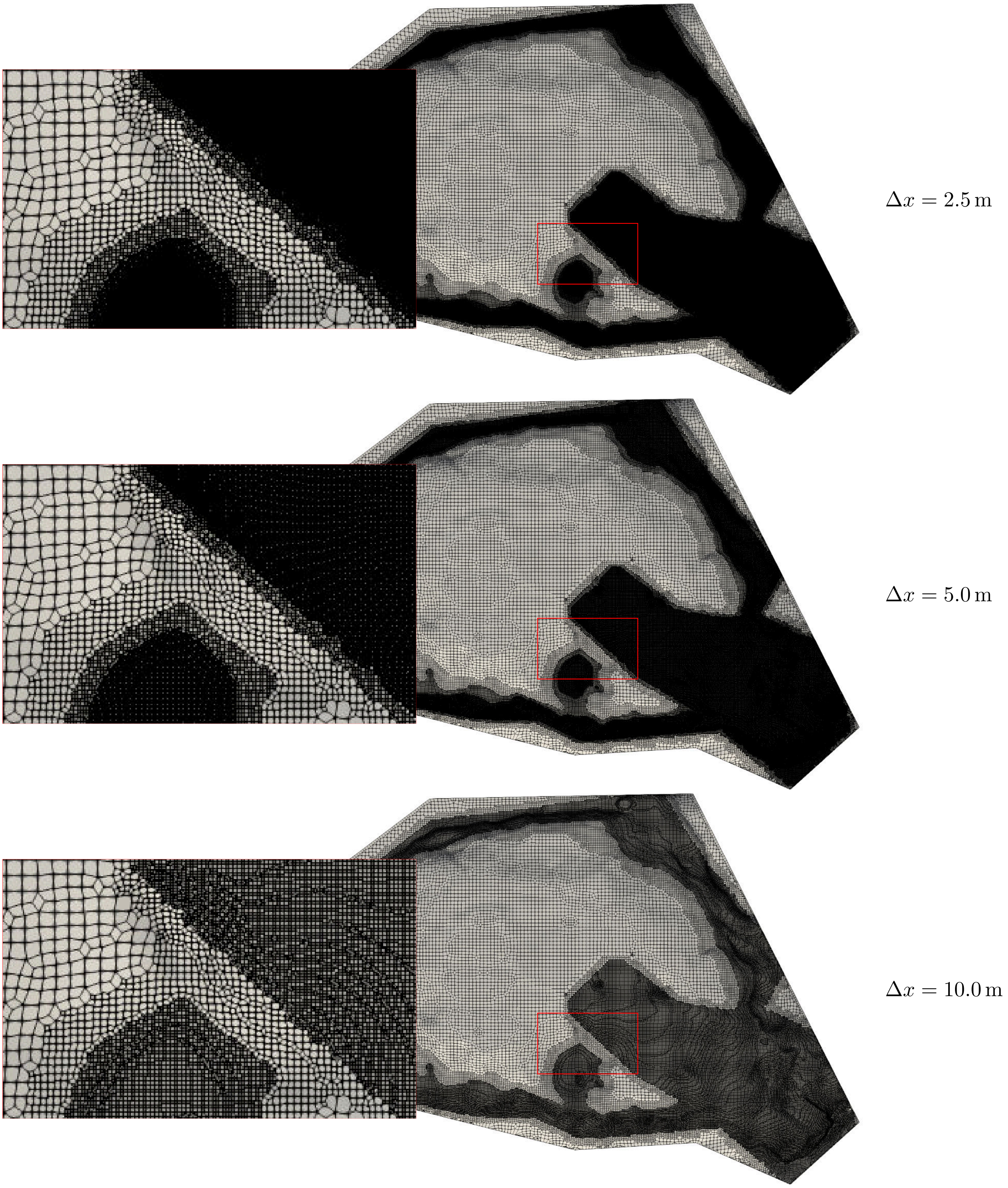

Figure 12: The four meshes used in the mesh refinement study. Only the lower boundary, representing the terrain is shown. The red sections are magnified on the left side.

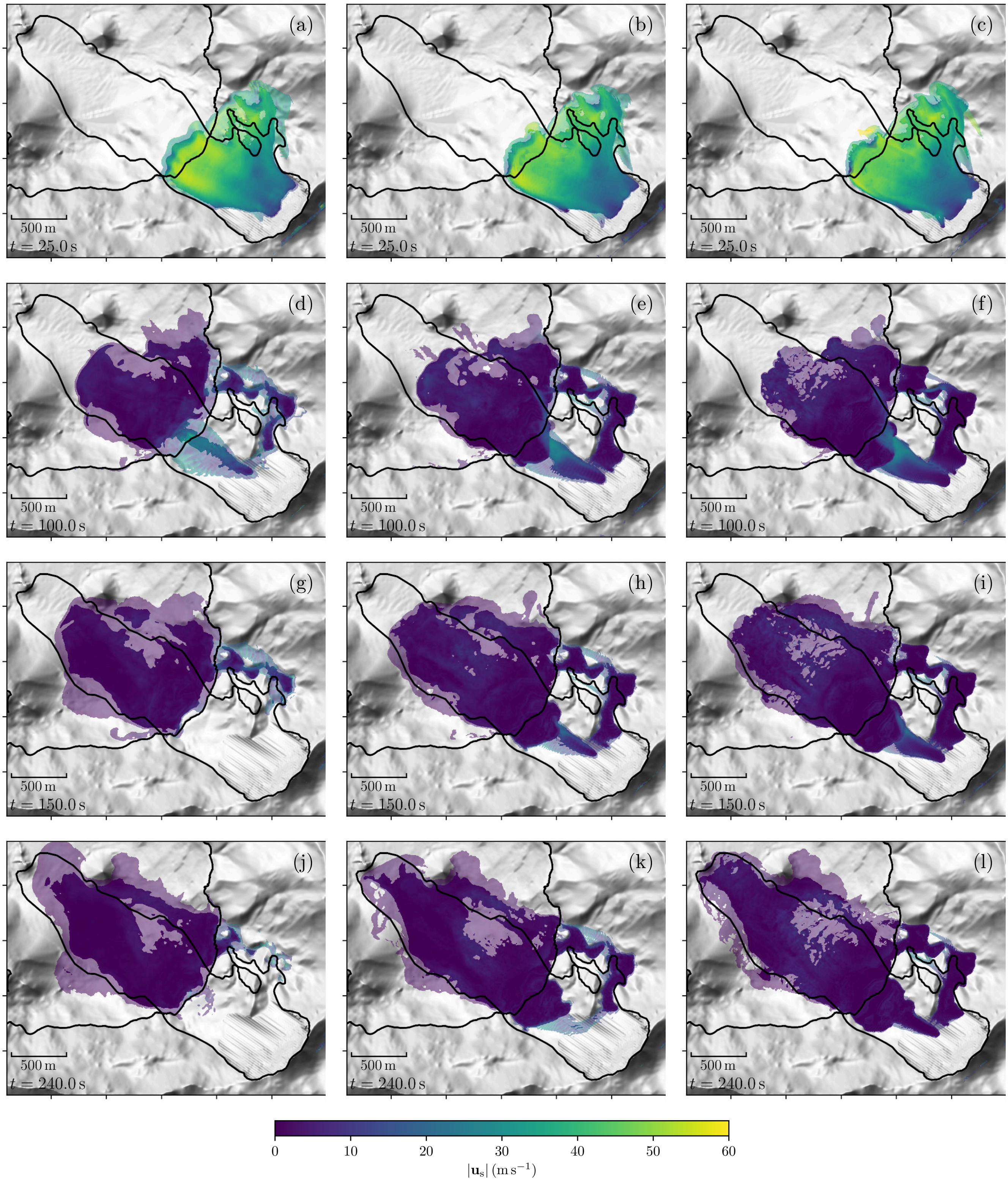

Figure 13: Comparison of the simulated slide at different levels of refinement: Left (a,d,g,j) 10 m, middle (b,e,h,k) 5 m and right (c,f,i,l) 2.5 m.

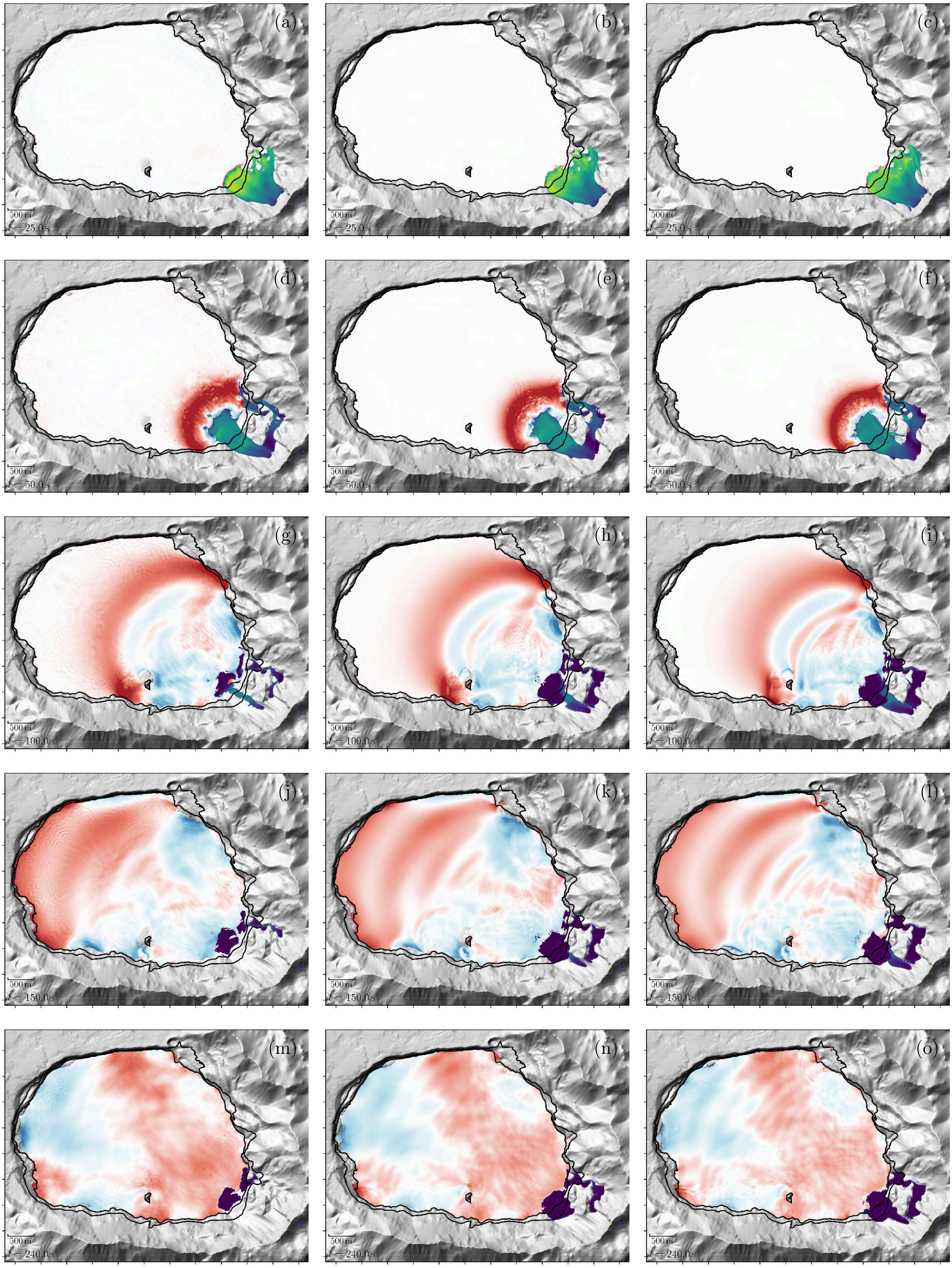

Figure 14: Comparison of the simulated wave at different levels of refinement: Left (a,d,g,j,m) 10 m, middle (b,e,h,k,n) 5 m and right (c,f,i,l,o) 2.5 m.

5 Sensitivity analysis of laboratory scale simulations

In this section, the sensitivity of the model to various material parameters is investigated. For this investigation, the laboratory experiment of Viroulet et al. [7] is repeated with modified material parameters. Material parameters are not changed individually but in groups to create reasonable scenarios (variation of critical packing density, variation of friction coefficients, etc.).

5.1 Geometry variation

Viroulet et al. [7] conducted multiple experiments with different slope angles ($\theta = 35°, 45°, 55°$) and slide masses ($m_0 = 1\,\text{kg}, 2\,\text{kg}, 3\,\text{kg}$) but the same material (parameters in the article). For a rigorous validation we simulated all cases with the same setup and parametrisation. The results are shown and compared to the experiments subsequently in terms of the (respective) wave signals.

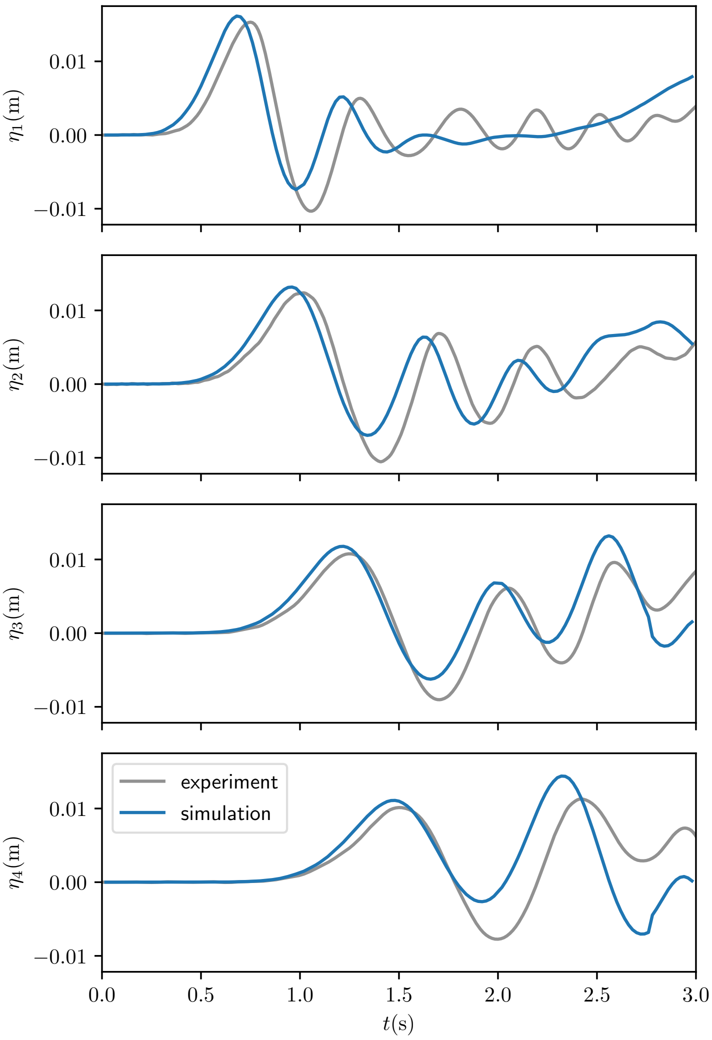

Figure 15: Experimental and numerical wave gauge measurements for the case with slope angle $\theta = 35°$ and 2 kg slide mass.

Table 6: Different error definitions for the wave signal at all gauges for the simulation with $\theta = 35°$ and $m_0 = 2\,\mathrm{kg}$.

gauge	L2 norm (mm)	mean error (mm)	maximum error (mm)
1	3.29	2.60	16.14
2	3.30	2.59	13.16
3	2.85	1.99	13.20
4	4.45	2.84	14.40

Table 7: Quantitative comparison between the experiment and simulation for $\theta = 35°$ and $m_0 = 2\,\mathrm{kg}$.

gauge	crest	a_{exp} (mm)	a_{sim} (mm)	Δa (mm)	$t_{\mathrm{arr,exp}}$ (s)	$t_{\mathrm{arr,sim}}$ (s)	Δt_{arr} (s)
1	1	15.3	16.1	0.826	0.8	0.7	0.072
1	2	5.0	5.2	0.187	1.3	1.2	0.084
2	1	12.4	13.2	0.777	1.0	1.0	0.060
2	2	6.9	6.4	0.498	1.7	1.6	0.078
3	1	10.8	11.8	1.011	1.3	1.2	0.030
3	2	6.1	6.8	0.701	2.1	2.0	0.066
4	1	10.1	11.1	0.966	1.5	1.5	0.024
4	2	11.3	14.4	3.112	2.4	2.3	0.102
gauge	trough	a_{exp} (mm)	a_{sim} (mm)	Δa (mm)	$t_{\mathrm{arr,exp}}$ (s)	$t_{\mathrm{arr,sim}}$ (s)	Δt_{arr} (s)
1	1	-10.3	-7.4	2.932	1.1	1.0	0.078
1	2	-2.8	-2.3	0.499	1.6	1.4	0.114
2	1	-10.5	-7.0	3.579	1.4	1.3	0.066
2	2	-5.3	-5.4	0.087	2.0	1.9	0.078
3	1	-9.0	-6.2	2.786	1.7	1.7	0.042
3	2	-4.0	-1.2	2.790	2.3	2.2	0.078
4	1	-7.7	-2.6	5.101	2.0	1.9	0.078
4	2	2.9	-7.0	9.934	2.7	2.7	0.006

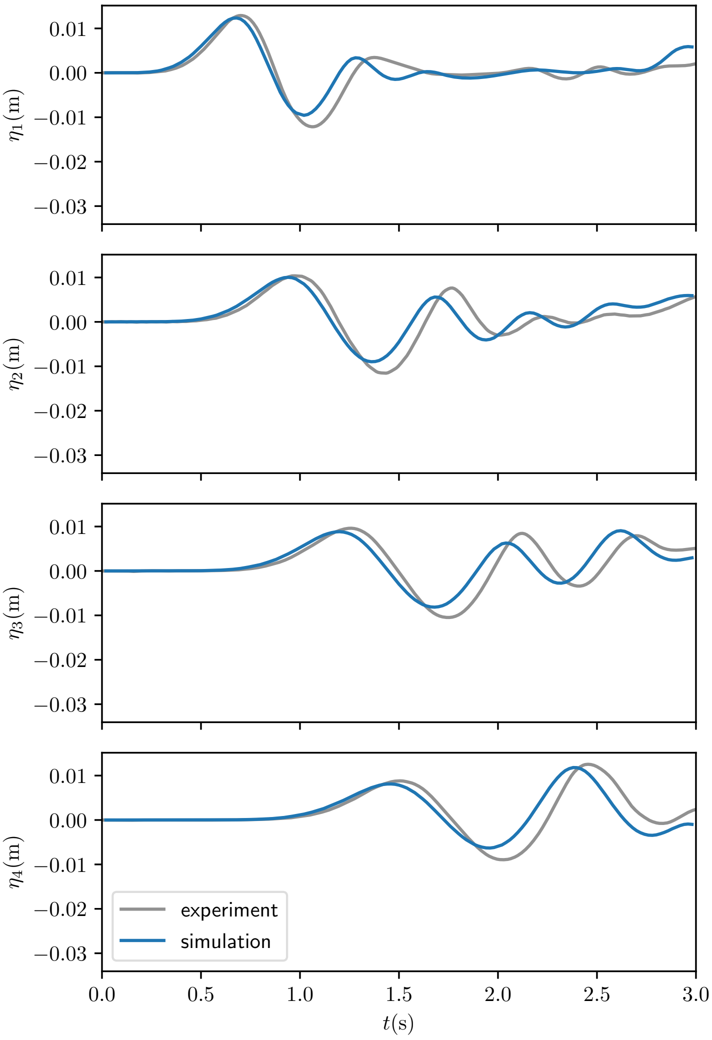

Figure 16: Experimental and numerical wave gauge measurements for the case with slope angle $\theta = 45°$ and 1 kg slide mass.

Table 8: Different error definitions for the wave signal at all gauges for the simulation with $\theta = 45^\circ$ and $m_0 = 1\,\text{kg}$.

gauge	L2 norm (mm)	mean error (mm)	maximum error (mm)
1	2.32	1.50	11.46
2	2.73	1.89	9.26
3	2.96	2.08	8.35
4	2.87	1.80	10.45

Table 9: Quantitative comparison between the experiment and simulation for $\theta = 45^\circ$ and $m_0 = 1\,\text{kg}$.

gauge	crest	a_{exp} (mm)	a_{sim} (mm)	Δa (mm)	$t_{\text{arr,exp}}$ (s)	$t_{\text{arr,sim}}$ (s)	Δt_{arr} (s)
1	1	12.9	11.5	1.440	0.7	0.7	0.024
1	2	3.5	4.1	0.641	1.4	1.3	0.114
2	1	10.4	9.3	1.116	1.0	0.9	0.030
2	2	7.6	5.6	2.053	1.8	1.7	0.090
3	1	9.6	8.2	1.457	1.3	1.2	0.054
3	2	8.5	5.8	2.666	2.1	2.0	0.102
4	1	8.8	7.5	1.357	1.5	1.5	0.048
4	2	12.5	10.5	2.088	2.5	2.4	0.078
gauge	trough	a_{exp} (mm)	a_{sim} (mm)	Δa (mm)	$t_{\text{arr,exp}}$ (s)	$t_{\text{arr,sim}}$ (s)	Δt_{arr} (s)
1	1	-12.1	-8.7	3.459	1.1	1.0	0.048
1	2	-1.4	-1.8	0.493	2.3	1.5	0.884
2	1	-11.6	-7.9	3.686	1.4	1.4	0.072
2	2	-3.0	-4.5	1.507	2.0	1.9	0.078
3	1	-10.5	-7.0	3.462	1.7	1.7	0.084
3	2	-3.4	-3.3	0.113	2.4	2.3	0.108
4	1	-8.9	-5.4	3.562	2.0	1.9	0.084

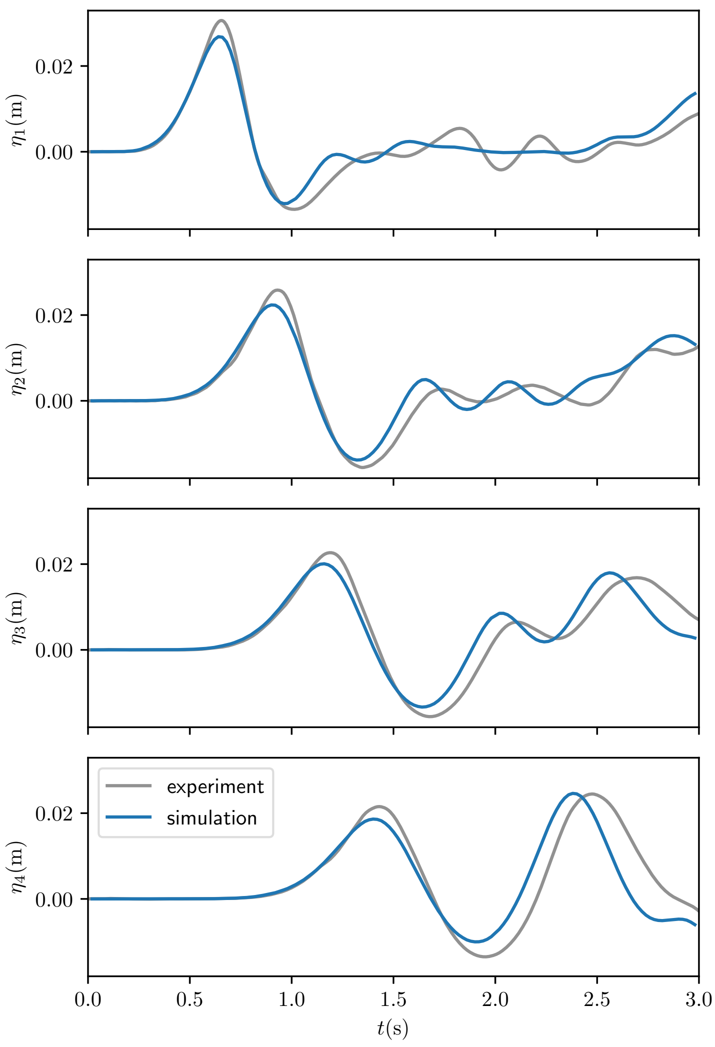

Figure 17: Experimental and numerical wave gauge measurements for the case with slope angle $\theta = 45°$ and 3 kg slide mass.

Table 10: Different error definitions for the wave signal at all gauges for the simulation with $\theta = 45°$ and $m_0 = 3\,\mathrm{kg}$.

gauge	L2 norm (mm)	mean error (mm)	maximum error (mm)
1	2.74	2.09	27.46
2	2.94	2.15	22.44
3	3.65	2.51	20.02
4	5.43	3.40	25.73

Table 11: Quantitative comparison between the experiment and simulation for $\theta = 45°$ and $m_0 = 3\,\mathrm{kg}$.

gauge	crest	a_{exp} (mm)	a_{sim} (mm)	Δa (mm)	$t_{\mathrm{arr,exp}}$ (s)	$t_{\mathrm{arr,sim}}$ (s)	Δt_{arr} (s)
1	1	30.6	27.5	3.152	0.7	0.6	0.012
1	2	-0.3	-0.8	0.473	1.4	1.2	0.246
2	1	25.8	22.4	3.371	0.9	0.9	0.030
2	2	2.8	6.4	3.604	1.7	1.6	0.090
3	1	22.7	20.0	2.645	1.2	1.2	0.030
3	2	6.5	10.1	3.620	2.1	2.0	0.084
4	1	21.5	18.5	3.047	1.4	1.4	0.030
4	2	24.5	25.7	1.262	2.5	2.4	0.096
gauge	trough	a_{exp} (mm)	a_{sim} (mm)	Δa (mm)	$t_{\mathrm{arr,exp}}$ (s)	$t_{\mathrm{arr,sim}}$ (s)	Δt_{arr} (s)
1	1	-13.4	-13.2	0.274	1.0	1.0	0.054
1	2	-1.1	-2.4	1.306	1.6	2.0	0.409
2	1	-15.6	-14.6	0.992	1.3	1.3	0.024
2	2	-0.2	-1.5	1.324	1.9	1.8	0.072
3	1	-15.6	-13.7	1.833	1.7	1.6	0.036
3	2	2.6	1.5	1.156	2.3	2.2	0.060
4	1	-13.5	-10.3	3.193	1.9	1.9	0.048

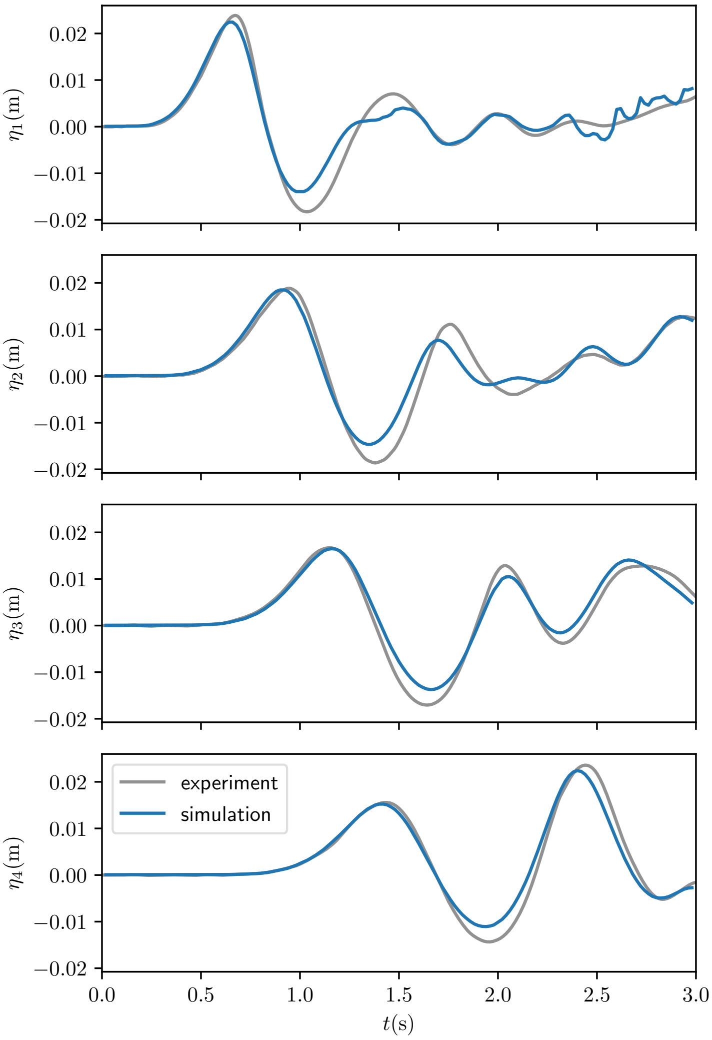

Figure 18: Experimental and numerical wave gauge measurements for the case with slope angle $\theta = 50^\circ$ and 2 kg slide mass.

Table 12: Different error definitions for the wave signal at all gauges for the simulation with $\theta = 50\,\mathrm{m}$ and $m_0 = 2\,\mathrm{kg}$.

gauge	L2 norm (mm)	mean error (mm)	maximum error (mm)
1	2.14	1.45	22.17
2	2.60	1.74	17.96
3	1.50	1.06	15.87
4	2.41	1.51	23.22

Table 13: Quantitative comparison between the experiment and simulation for $\theta = 50°$ and $m_0 = 2\,\mathrm{kg}$.

gauge	crest	a_{exp} (mm)	a_{sim} (mm)	Δa (mm)	$t_{\mathrm{arr,exp}}$ (s)	$t_{\mathrm{arr,sim}}$ (s)	Δt_{arr} (s)
1	1	23.9	22.2	1.691	0.7	0.7	0.018
1	2	7.0	5.9	1.135	1.5	1.5	0.030
2	1	18.9	18.0	0.896	0.9	0.9	0.042
2	2	11.1	10.4	0.726	1.8	1.7	0.066
3	1	16.7	15.9	0.828	1.1	1.2	0.012
3	2	12.9	12.8	0.027	2.0	2.0	0.000
4	1	15.6	14.5	1.096	1.4	1.4	0.012
4	2	23.6	23.2	0.363	2.4	2.4	0.060
gauge	trough	a_{exp} (mm)	a_{sim} (mm)	Δa (mm)	$t_{\mathrm{arr,exp}}$ (s)	$t_{\mathrm{arr,sim}}$ (s)	Δt_{arr} (s)
1	1	-18.3	-15.6	2.655	1.0	1.0	0.054
1	2	-3.9	-5.6	1.757	1.8	1.7	0.024
2	1	-18.6	-15.5	3.141	1.4	1.3	0.036
2	2	-3.9	-2.8	1.183	2.1	2.0	0.126
3	1	-17.1	-14.0	3.036	1.6	1.7	0.018
3	2	-3.8	-3.5	0.269	2.3	2.3	0.006
4	1	-14.4	-11.2	3.125	2.0	1.9	0.018

5.2 Friction coefficients

Friction coefficients μ_s and μ_d are changed together to simulate a low and high frictional material. The slide is respectively faster/slower and the wave is higher/lower.

Table 14: Modified friction coefficients and the respective errors in the first wave crest at the first gauge and the L2 error.

scenario	μ_s	μ_d	Δa_1	L2 Error η_1
low friction	0.24	0.49	4.949 mm	3.41 mm
standard	0.39	0.64	0.082 mm	2.07 mm
high friction	0.54	0.79	4.255 mm	3.40 mm

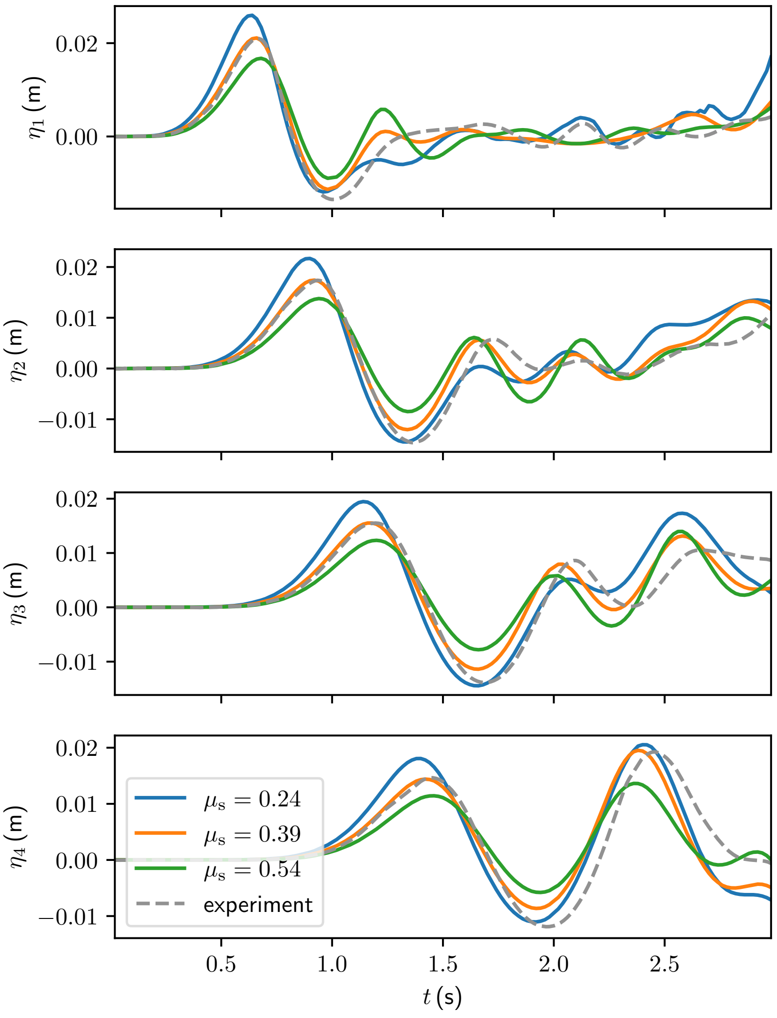

Figure 19: Wave gauges for a variation of friction coefficients.

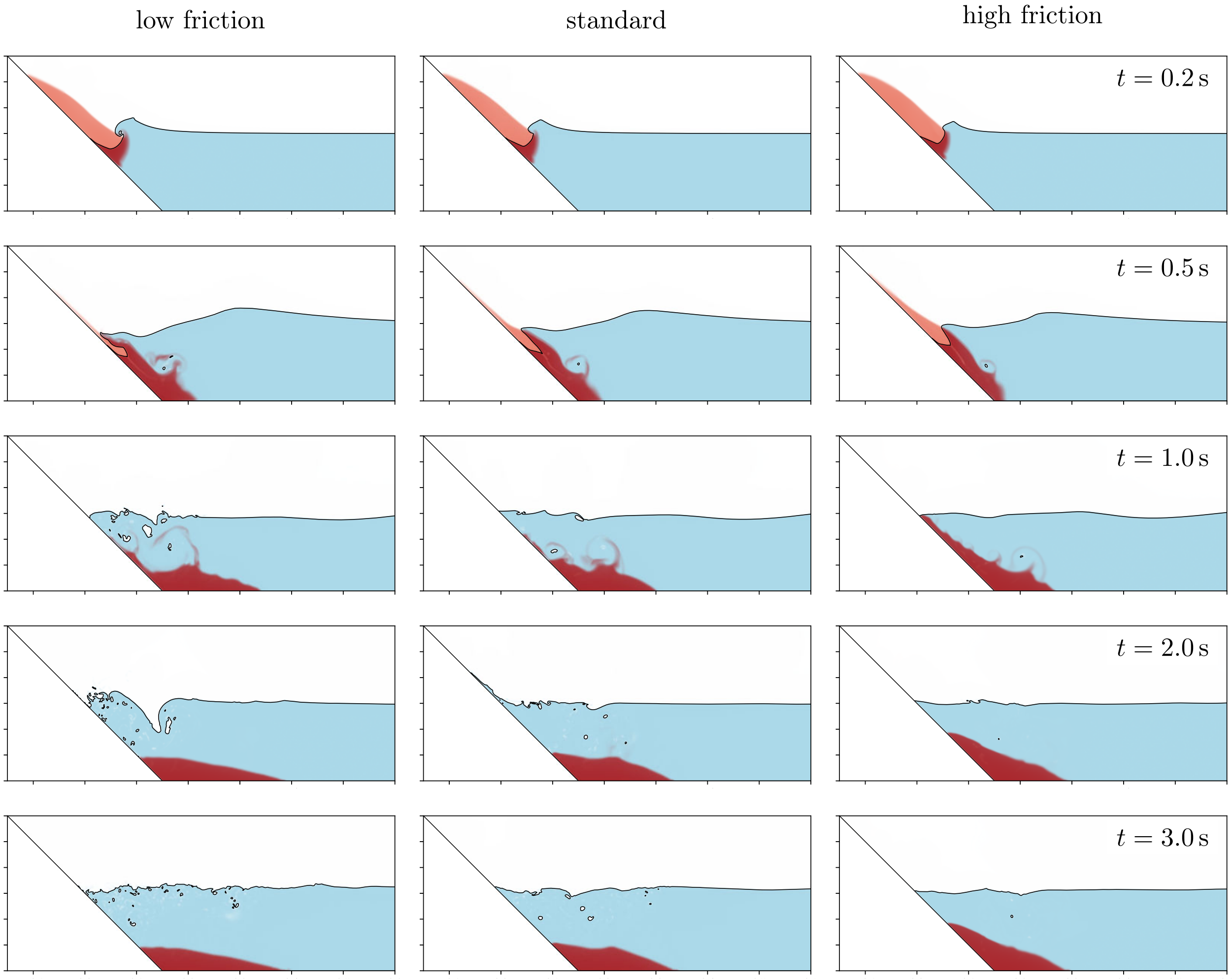

Figure 20: Difference in landslide and wave dynamics for a variation of friction coefficients.

5.3 Limiting packing densities

Limiting packing densities ϕ_{rlp} and μ_{rcp} are changed together to simulate a material that allows lower/higher packing densities. The total granular mass is constant and the lower packing density moves the centre of gravity of the initial pile upwards. This leads to a higher potential energy in the initial granular mass and thus a higher wave for looser material. The opposite is the case for higher packing densities and the result is a lower wave.

Table 15: Modified limiting packing densities and the respective errors in the first wave crest at the first gauge and the L2 error

scenario	ϕ_{rlp}	ϕ_{rcp}	Δa_1	L2 Error η_1
low packing limits	0.43	0.53	1.414 mm	2.79 mm
standard	0.53	0.63	0.082 mm	2.07 mm
high packing limits	0.63	0.73	2.817 mm	3.34 mm

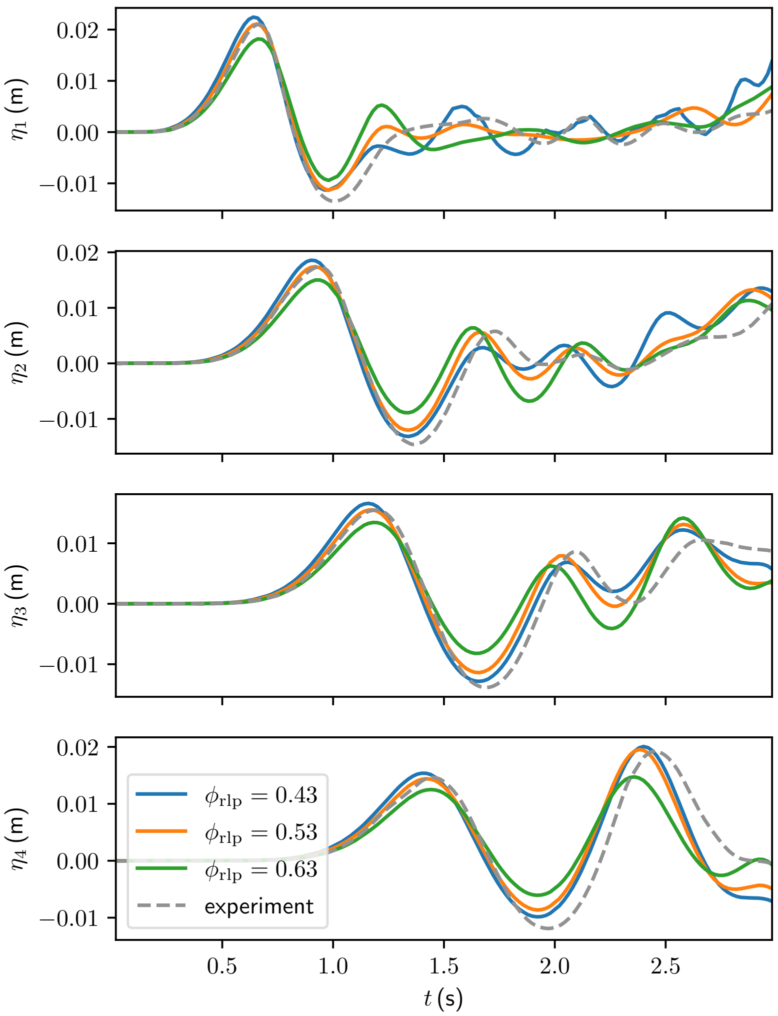

Figure 21: Wave gauges for a variation of the limiting packing densities.

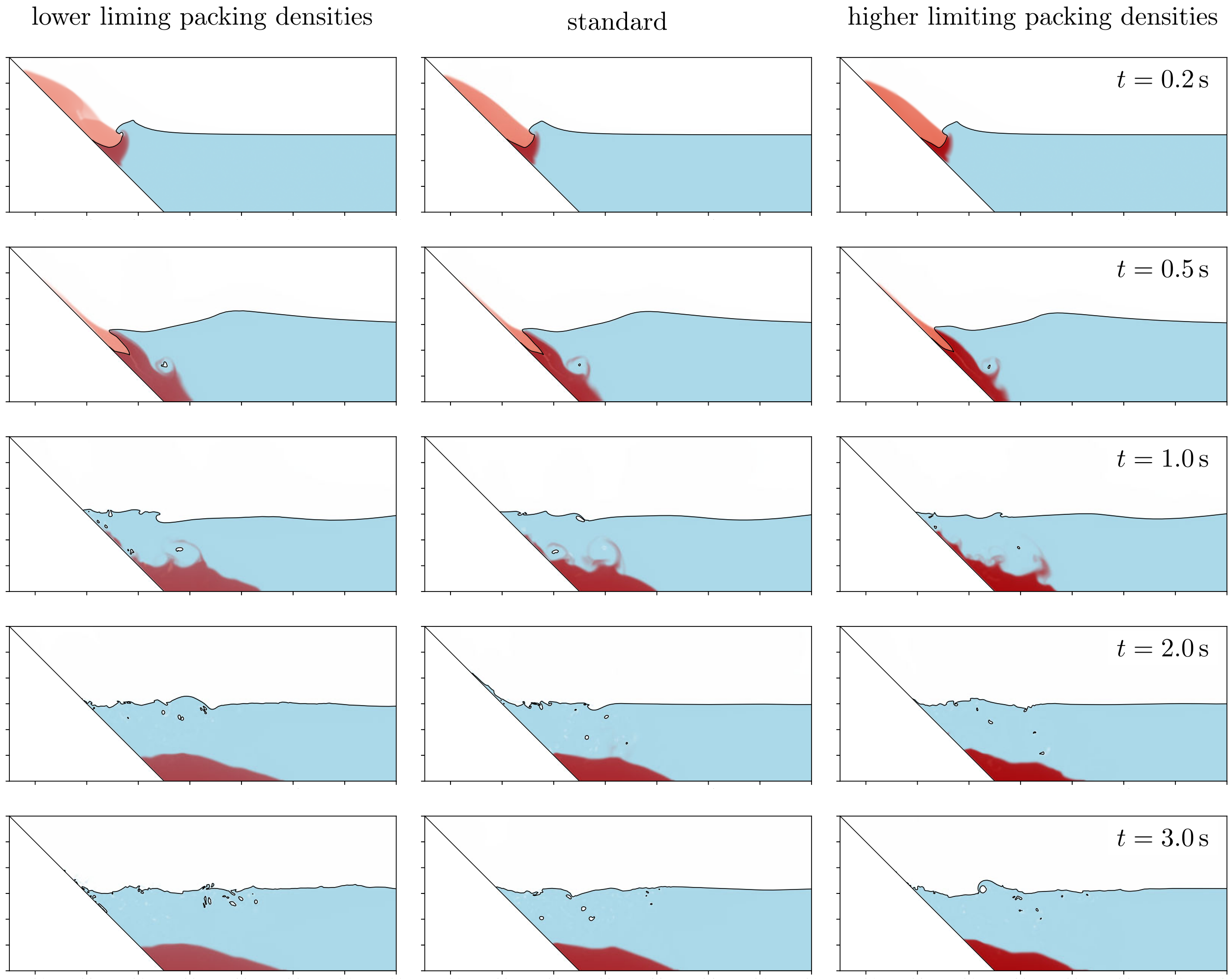

Figure 22: Difference in landslide and wave dynamics for a variation of the limiting packing densities.

5.4 Dynamic dilatancy

The dynamic dilatancy factor is modified between 0.025 and 0.075. Higher values showed to be unstable. The influence of this factor is small.

Table 16: Modified dynamic dilatancy factor and the respective errors in the first wave crest at the first gauge and the L2 error.

scenario	$\Delta\phi$	Δa_1	L2 Error η_1
low dilatancy	0.025	0.210 mm	2.76 mm
standard	0.05	0.082 mm	2.07 mm
high dilatancy	0.075	0.256 mm	2.03 mm

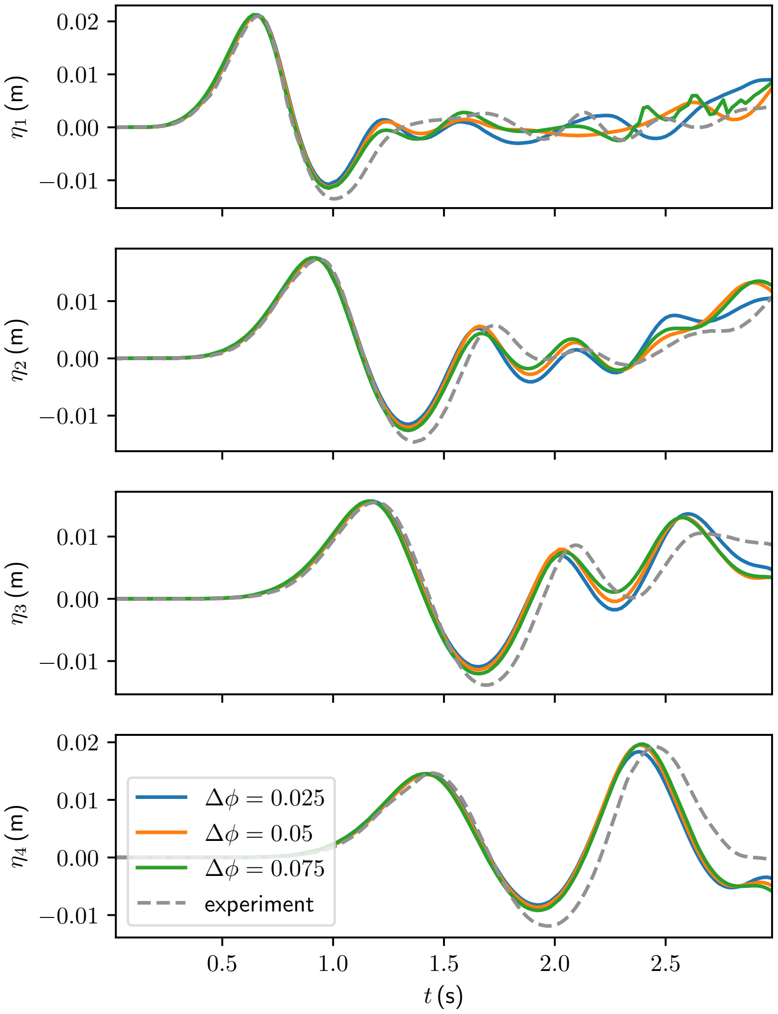

Figure 23: Wave gauges for a variation of the dynamic dilatancy factor.

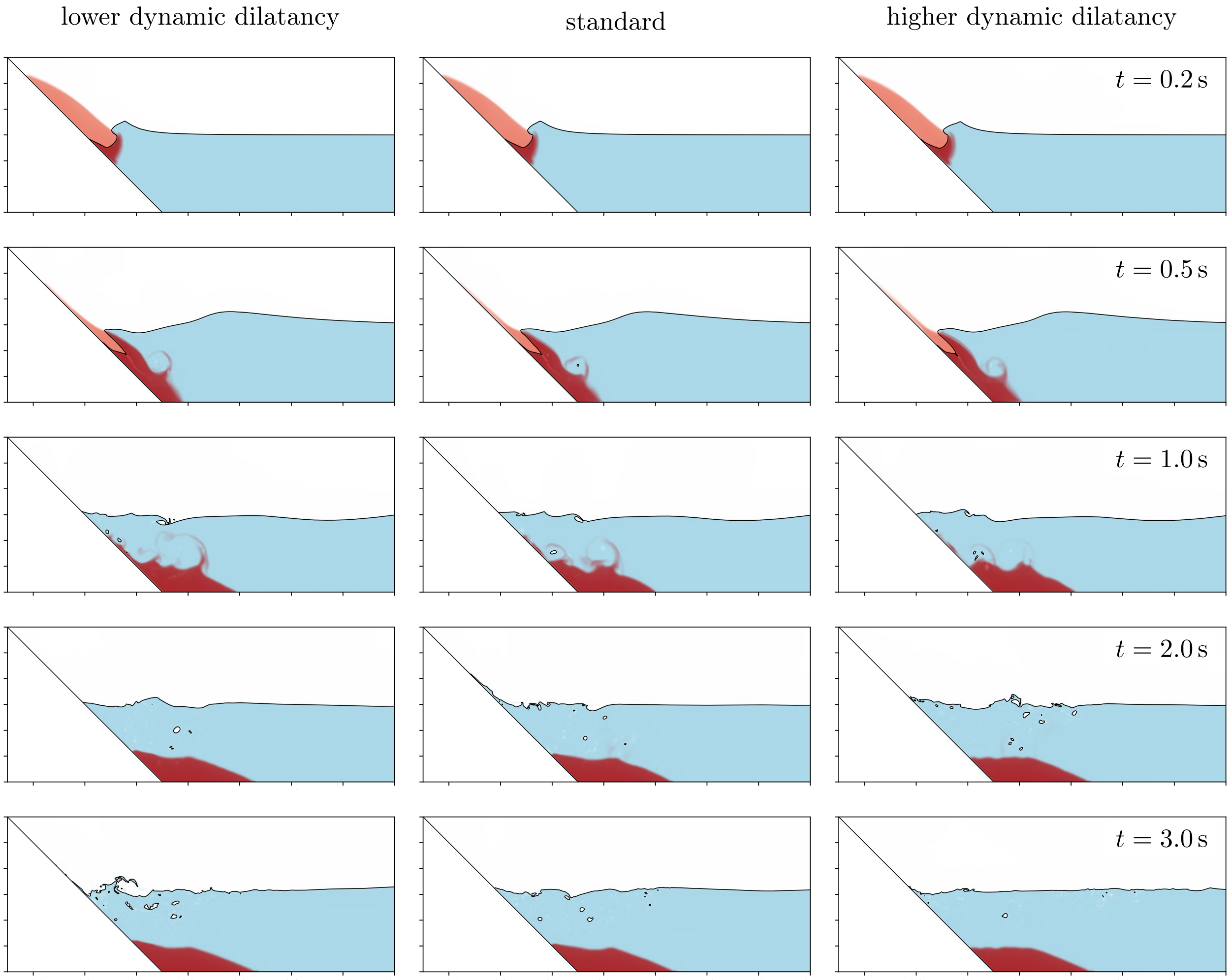

Figure 24: Difference in landslide and wave dynamics for a variation of the dynamic dilatancy factor.

5.5 Permeability

To investigate the effects of a variable permeability, the grain diameter was varied. In order to exclude influences of the rheology, I_0 and $\Delta\phi$ have been adjusted to counteract the change of the grain diameter in the $\mu(I)$, $\phi(I)$-rheology.

The influence of the permeability on the generated wave is small but the influence on the slide kinematics is clearly visible. The lower permeability of the slide leads to a bigger resistance of the water reservoir and the slide is under water respectively slower and the runout shorter. The opposite is the case for a higher permeability and the slide is faster under water and runs further. Although the slide differs in velocity, the respective difference in displaced water leads to a similar wave height.

Table 17: Modified permeability (trough the particle diameter) and the respective errors in the first wave crest at the first gauge and the L2 error.

scenario	d	I_0	$\Delta\phi$	Δa_1	L2 Error η_1
low permeability	0.001 m	0.075	0.20	0.311 mm	2.04 mm
standard	0.004 m	0.30	0.05	0.082 mm	2.07 mm
high permeability	0.016 m	1.20	0.0125	0.968 mm	3.25 mm

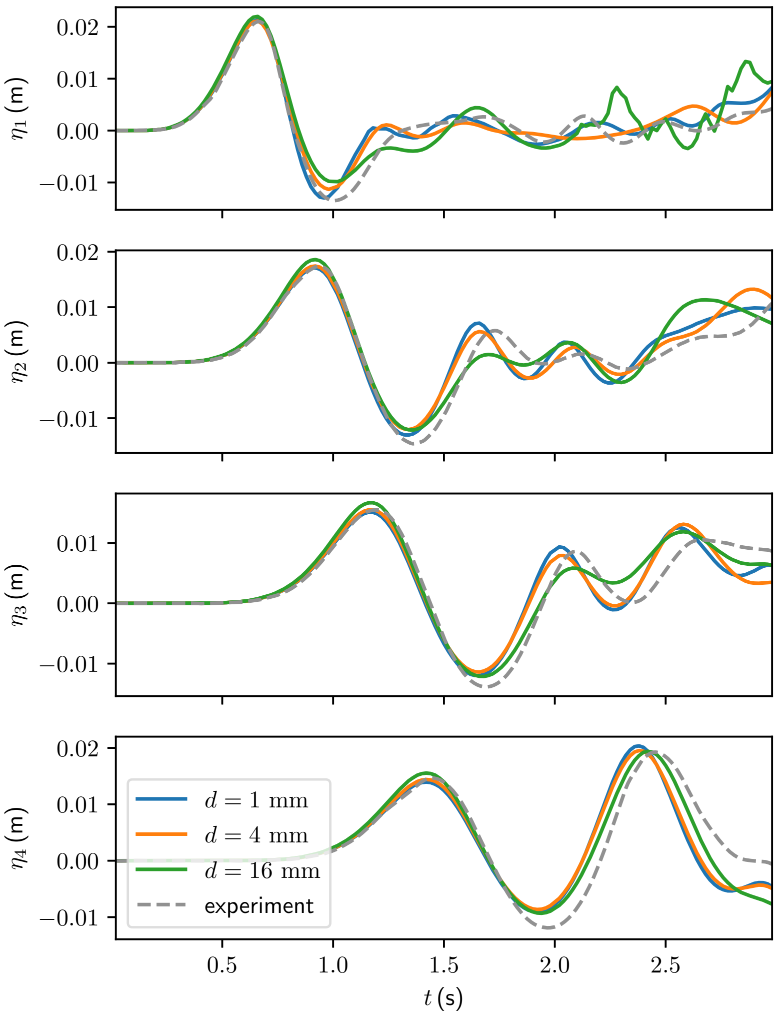

Figure 25: Wave gauges for a variation in the permeability.

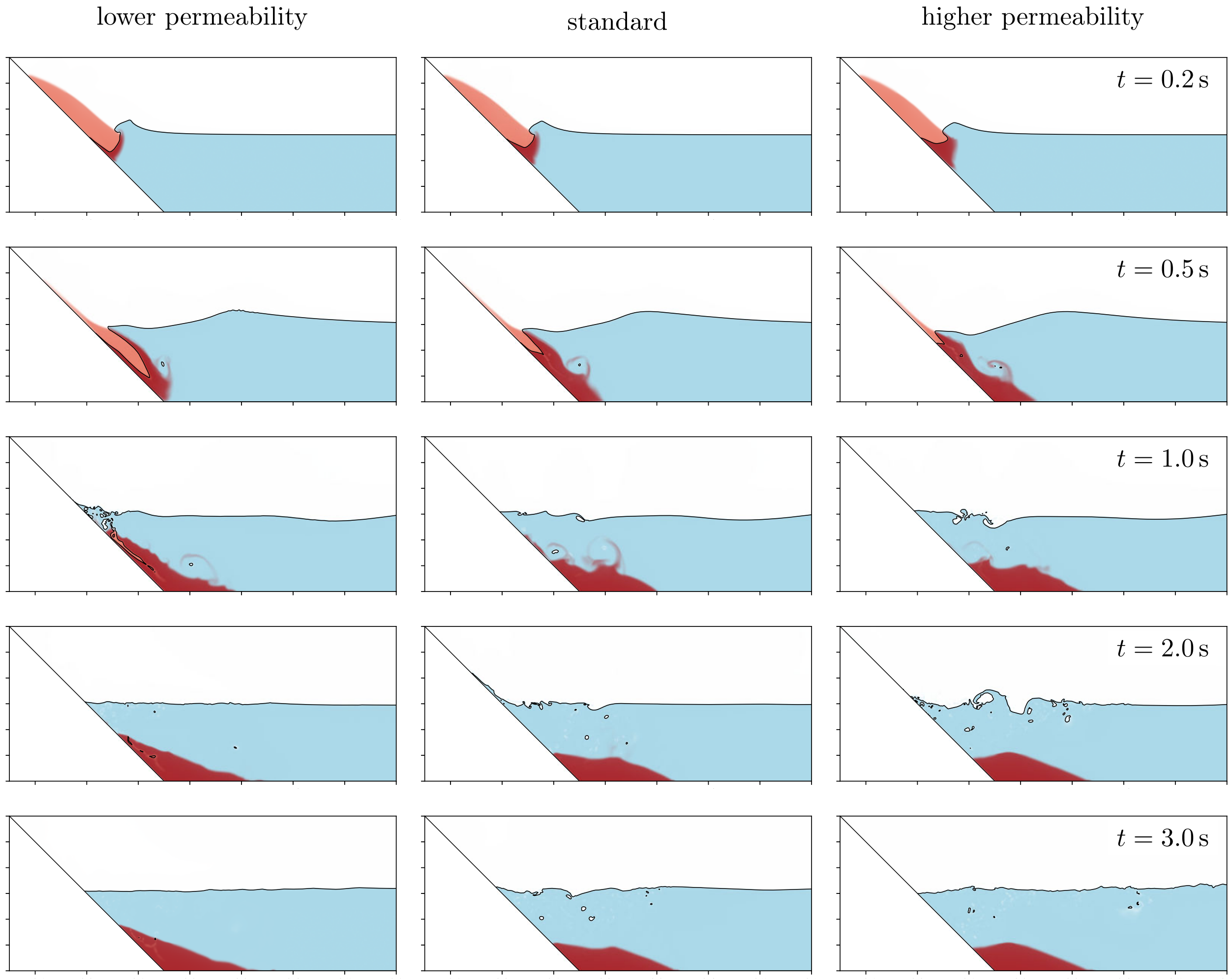

Figure 26: Difference in landslide and wave dynamics for a variation in the permeability.

5.6 Particle diameter

Changing the particle diameter has many consequences, e.g. variations in dynamic friction, dilatancy and permeability. A particle diameter of 16 mm leads to unstable simulations, if the material parameters I_0 and $\Delta\phi$ are not adjusted accordingly. We choose an upper limit of 8 mm. The results are shown in Figs. 27 and 28. The slide behaves different due to the changed permeability and the rheology. The results are comparable to the changed permeability (see above) and an additionally changed friction.

Table 18: Modified particle diameter and the respective errors in the first wave crest at the first gauge and the L2 error.

scenario	d	Δa_1	L2 Error η_1
low diameter	0.001 m	4.277 mm	2.54 mm
standard	0.004 m	0.082 mm	2.07 mm
high diameter	0.008 m	0.559 mm	2.72 mm

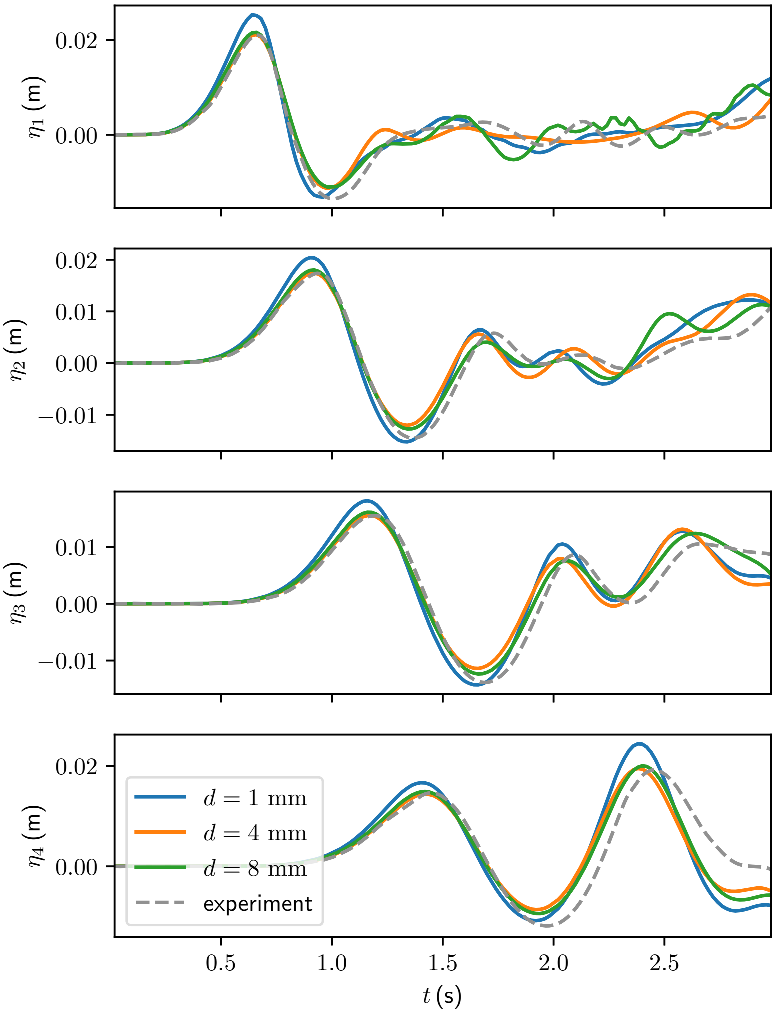

Figure 27: Wave gauges for a variation in particle diameter.

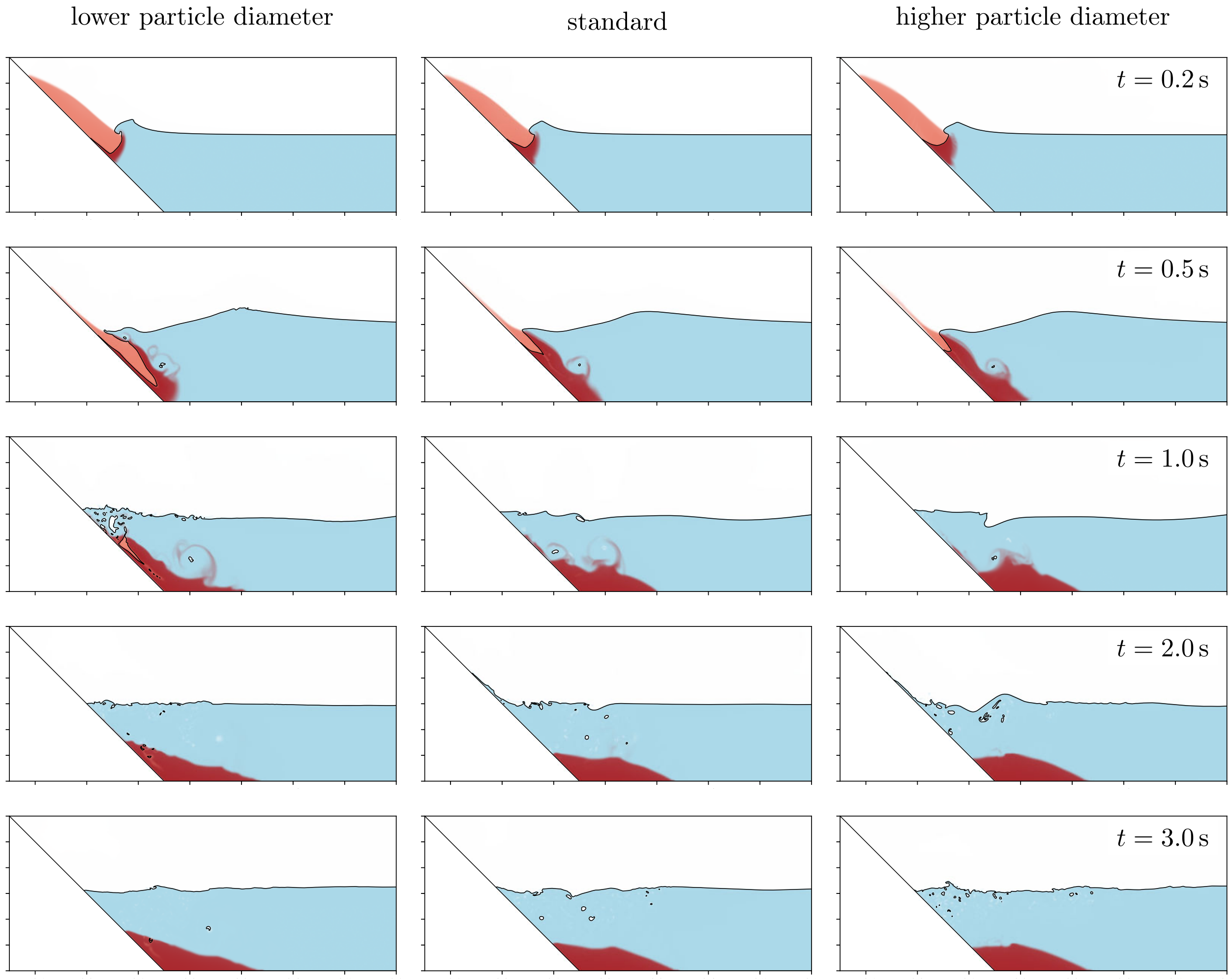

Figure 28: Difference in landslide and wave dynamics for a variation of the particle diameter.

5.7 Grain density

The grain density was changed while keeping the limiting packing densities and the scaling factor a constant. The packing density of the pile had to be initialized in relation to the changed grain density and a higher density lead to a higher initial packing density and a lower centre of gravity. The slide had thus less potential energy and the wave height was lower. The opposite was observed with a lower density.

Table 19: Modified grain density and the respective errors in the first wave crest at the first gauge and the L2 error.

scenario	ρ_s	Δa_1	L2 Error η_1
low density	$2\,000\,\mathrm{kg\,m^{-3}}$	2.241 mm	2.89 mm
standard	$2\,500\,\mathrm{kg\,m^{-3}}$	0.082 mm	2.07 mm
high density	$3\,000\,\mathrm{kg\,m^{-3}}$	2.381 mm	2.13 mm

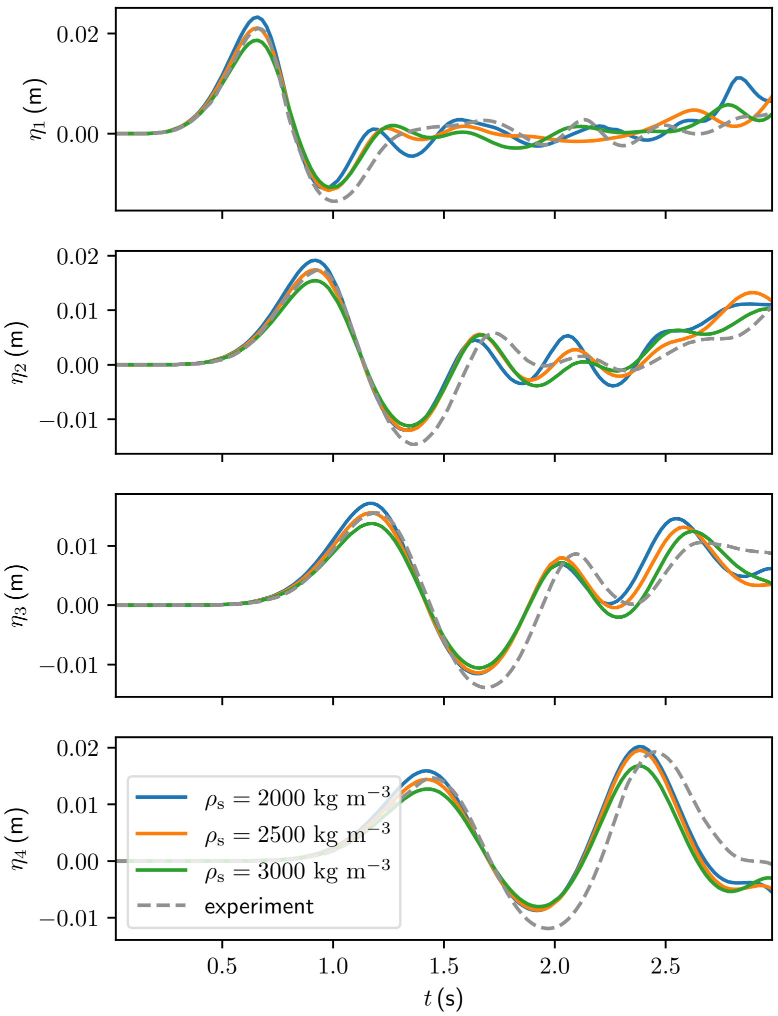

Figure 29: Wave gauges for a variation of the grain density.

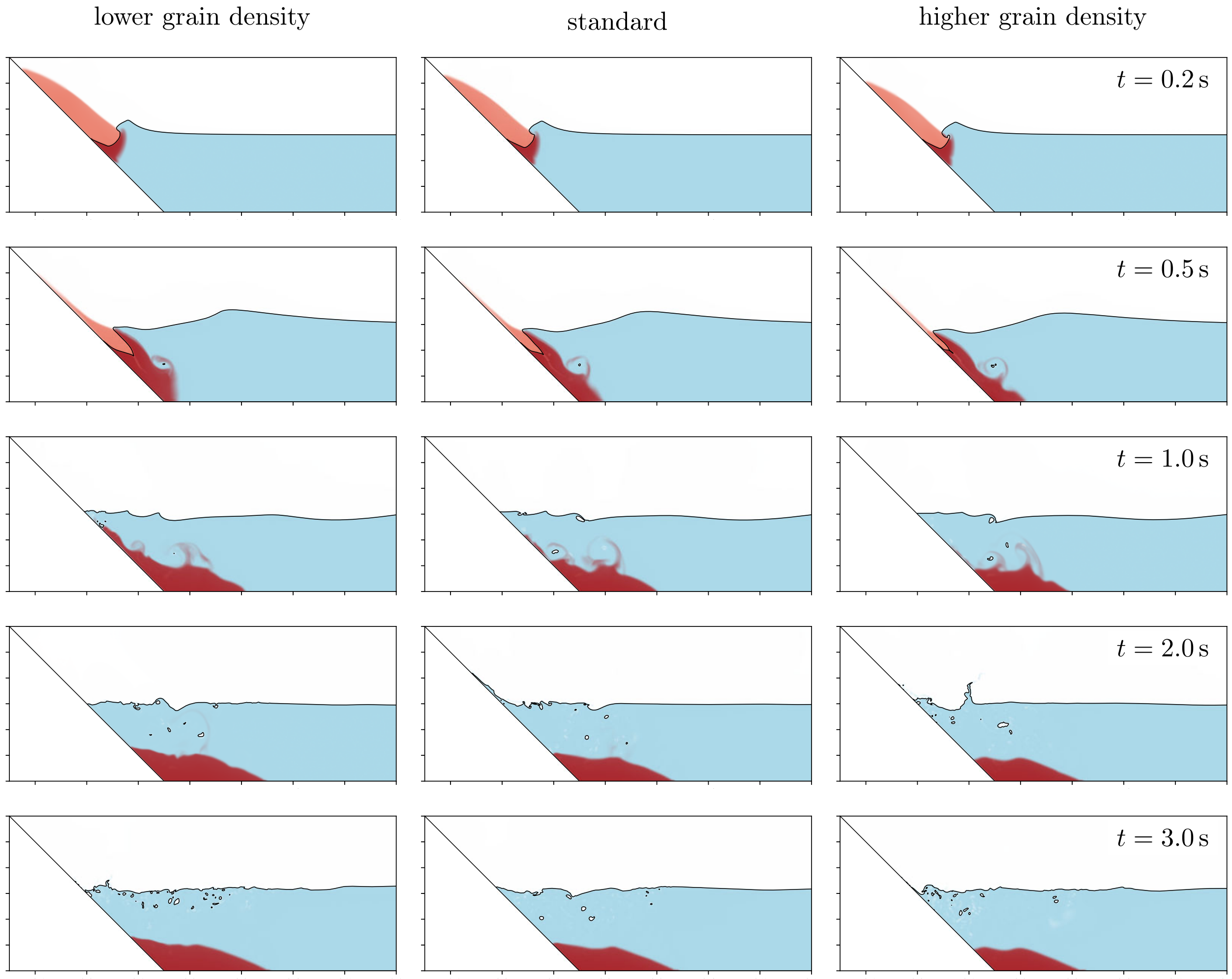

Figure 30: Difference in landslide and wave dynamics for a variation of the grain density.

5.8 The effect of a subgrid turbulence model

The Reynolds number is estimated as $10^5 - 10^6$ for the small case experiment and as $10^8 - 10^{10}$ for the real scale case, which indicates, that some turbulent motion cannot be resolved by the numerical method and has to be modelled.

Turbulence modelling is a very complex field and multiple phases, some of which are porous, makes this topic even more complicated. In fact, there is no generally agreed method to include subgrid turbulence and this topic is highly discussed and under investigation. Here we apply the simplest turbulence model with its standard parameters to estimate the effect a turbulence model would have on the simulations, similar to Rauter et al. [5]. All intrinsic phase viscosities are supplemented with the turbulent viscosity ν_t,

$$\nu_{\mathrm{eff},i} = \nu_i + \nu_\mathrm{t}, \tag{44}$$

which we calculate following the k-ε turbulence model as

$$\nu_\mathrm{t} = \overline{\rho}\, C_\nu \frac{k^2}{\varepsilon}. \tag{45}$$

Notably, this applies also to the granular phase viscosity ν_g, however, which is much higher than the turbulent viscosity at all times. The turbulent kinetic energy $k(\mathbf{x}, t)$ and rate of dissipation $\varepsilon(\mathbf{x}, t)$ follow as

$$\frac{\partial \overline{\rho}\, k}{\partial t} + \nabla\cdot(\overline{\rho}\, k\, \overline{\mathbf{u}}) = \nabla\cdot\left(\overline{\rho}\left(\frac{\nu_t}{\sigma_k} + \overline{\nu}\right)\nabla k\right) + \overline{\rho}\,\nu_\mathrm{t}\,(2\,\overline{\mathbf{D}}) : \nabla\,\overline{\mathbf{u}} - \overline{\rho}\,\varepsilon, \tag{46}$$

$$\frac{\partial \overline{\rho}\, \varepsilon}{\partial t} + \nabla\cdot(\overline{\rho}\, \varepsilon\, \overline{\mathbf{u}}) = \nabla\cdot\left(\overline{\rho}\left(\frac{\nu_\mathrm{t}}{\sigma_\varepsilon} + \overline{\nu}\right)\nabla \varepsilon\right) + \overline{\rho}\,\frac{C_1\,\nu_\mathrm{t}\,\varepsilon}{k}(2\,\overline{\mathbf{D}}) : \nabla\,\overline{\mathbf{u}} - C_2\,\overline{\rho}\frac{\varepsilon^2}{k}. \tag{47}$$

Standard parameters, $C_\nu = 0.09$, $\sigma_k = 1.0$, $\sigma_\varepsilon = 1.3$, $C_1 = 1.44$, $C_2 = 1.92$ [8], have been applied for this test and k and ε were initialized to very small values.

The sub grid turbulence has the following effects: The macroscopic wave is stronger damped and after the first crest smoother than the simulation without the turbulence model and the experiment, especially at the first wave gauge. All other gauges are very similar between simulations with and without the turbulence model. The wave signal at the second gauge is slightly more accurate with the turbulence model. The impact area is less turbulent and the counter clockwise eddy in the impact zone is less prominent with the turbulence model. As the eddy is less prominent, the deposition is slightly higher towards on the inclined slope and this represents the experiment more accurately.

We conclude that the impact zone and the deposition match the experiment better if a sub grid turbulence model is applied. However, the influence on the macroscopic wave can be slightly negative and dampen the wave too strongly, as shown by previous studies [e.g. 9]. For the real scale simulation, we decided to use no sub grid turbulence model although the Reynolds number is very high.

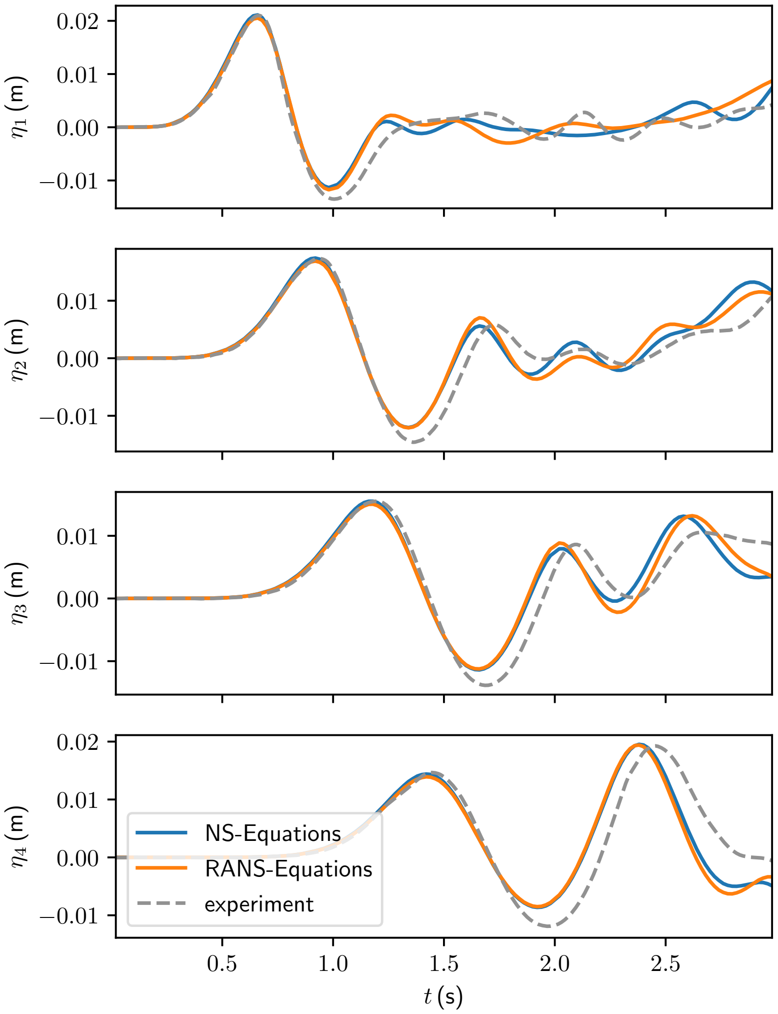

Figure 31: Effect of the subgrid turbulence model: Wave gauges for the simulations with and without a subgrid turbulence model.

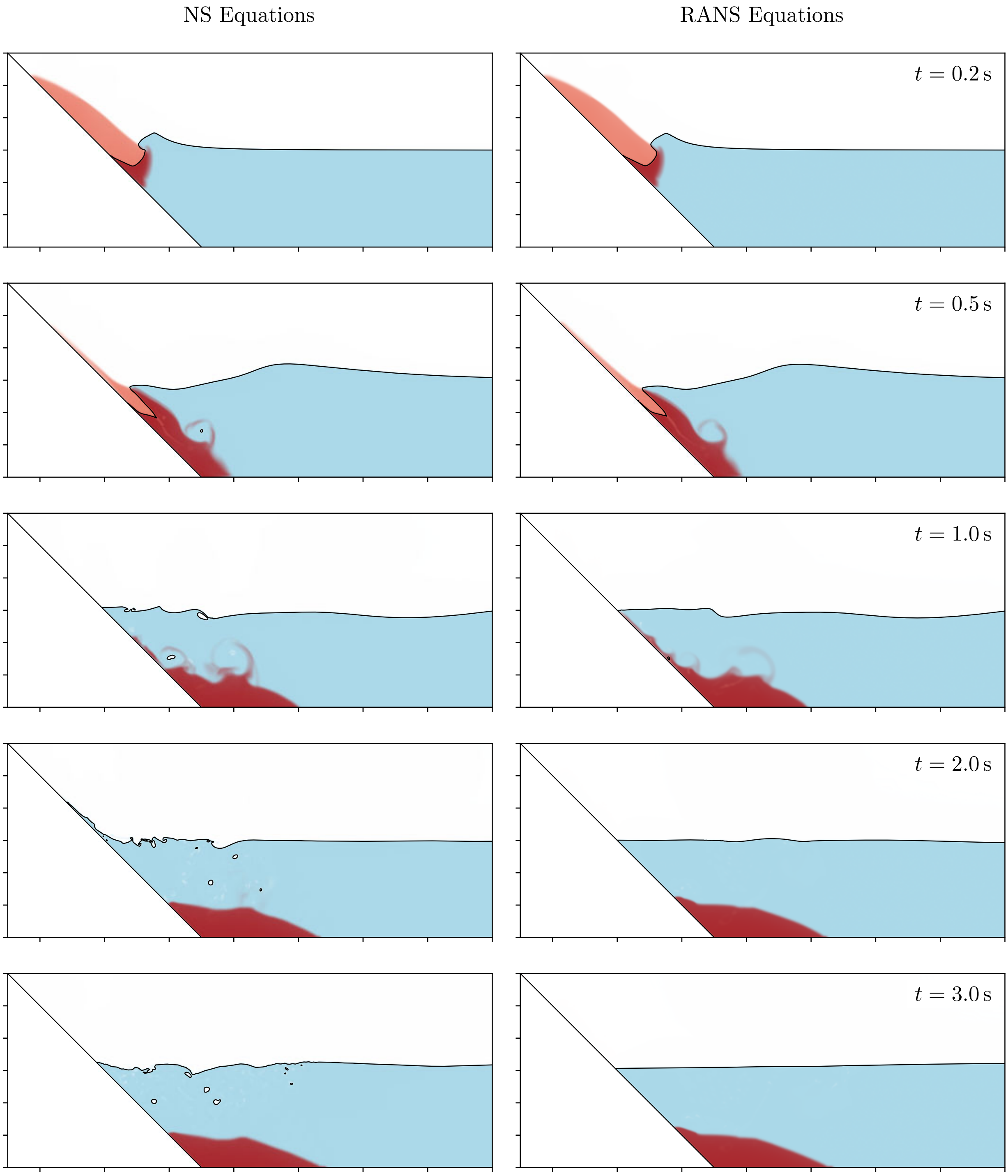

Figure 32: Effect of the subgrid turbulence model: Difference in landslide and wave dynamics for the simulations with and without a subgrid turbulence model.

6 Approximation of the experiment with a two-dimensional model

The experiment of Viroulet et al. [7] was designed in such a way that it can be approximated by two-dimensional models (vertical and longitudinal axes). The side walls are smooth and all initial material is uniformly distributed along the width of the tank. It is assumed that the shear traction on side walls can be neglected, which is the requirement to reasonably approximate the case with a two-dimensional width-averaged simulation. The slide and wave front in the experiments formed a straight line (see Fig. 33), which is an indicator that a two-dimensional approximation might be sufficient.

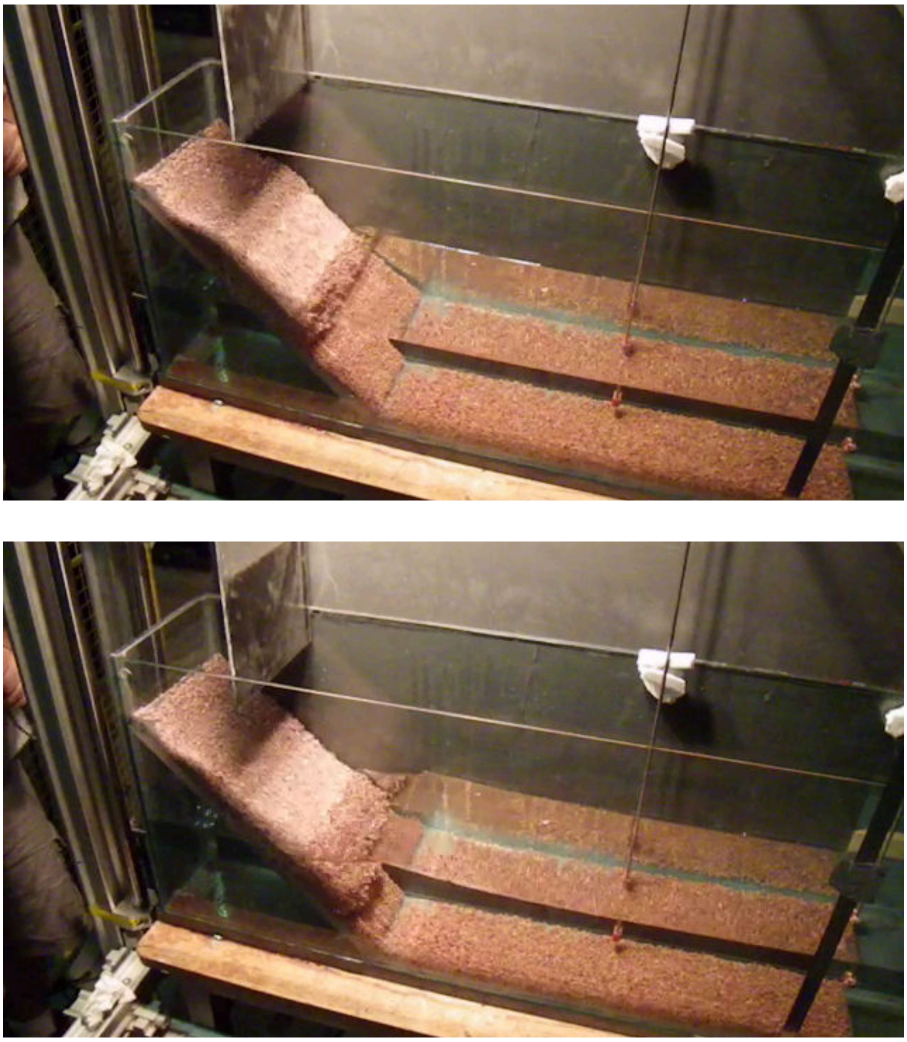

Figure 33: Perspective view on the laboratory experiment at two different points in time.

We further investigated this issue by running a full three-dimensional simulation of the laboratory experiment and by comparing the results to the experiment and the two-dimensional approximation. The wall friction angle between grains and the smooth walls was not measured during the experiments. We assumed a value of 10°. The mesh resolution (see Fig. 34) is reduced in comparison to the two-dimensional case, especially in the far field, to keep the computational cost low. A perspective view of the simulation is shown in Fig. 35. As in the experiment, the slide and wave front is close to a straight line. Virtual wave gauges were positioned in the middle of the tank and in a distance of 1 cm from the side wall to highlight the effect of the side walls on the wave. The resulting wave signals are shown in Fig. 36 and compared with the same signals from the two-dimensional simulation and the experiment. Note that the post-processing software failed to find the free surface for gauge 3 near the wall over 2 short periods. The wave signals between centre and border are basically the same and very similar to the wave signal from the two-dimensional case. All results fit the experiment, where the wave gauges where installed in the middle, fairly well. The small difference is below the numerical uncertainty (compare mesh refinement study) and thus likely related to the mesh resolution.

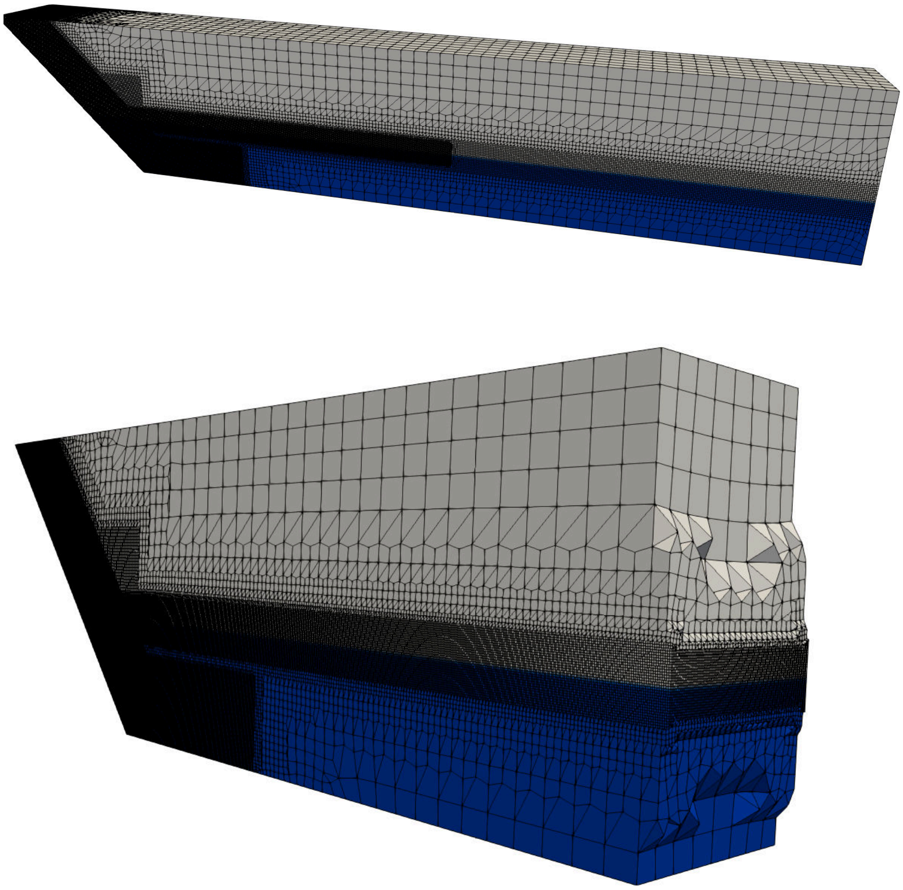

Figure 34: The three-dimeninsional mesh for the laboratory experiment simulation.

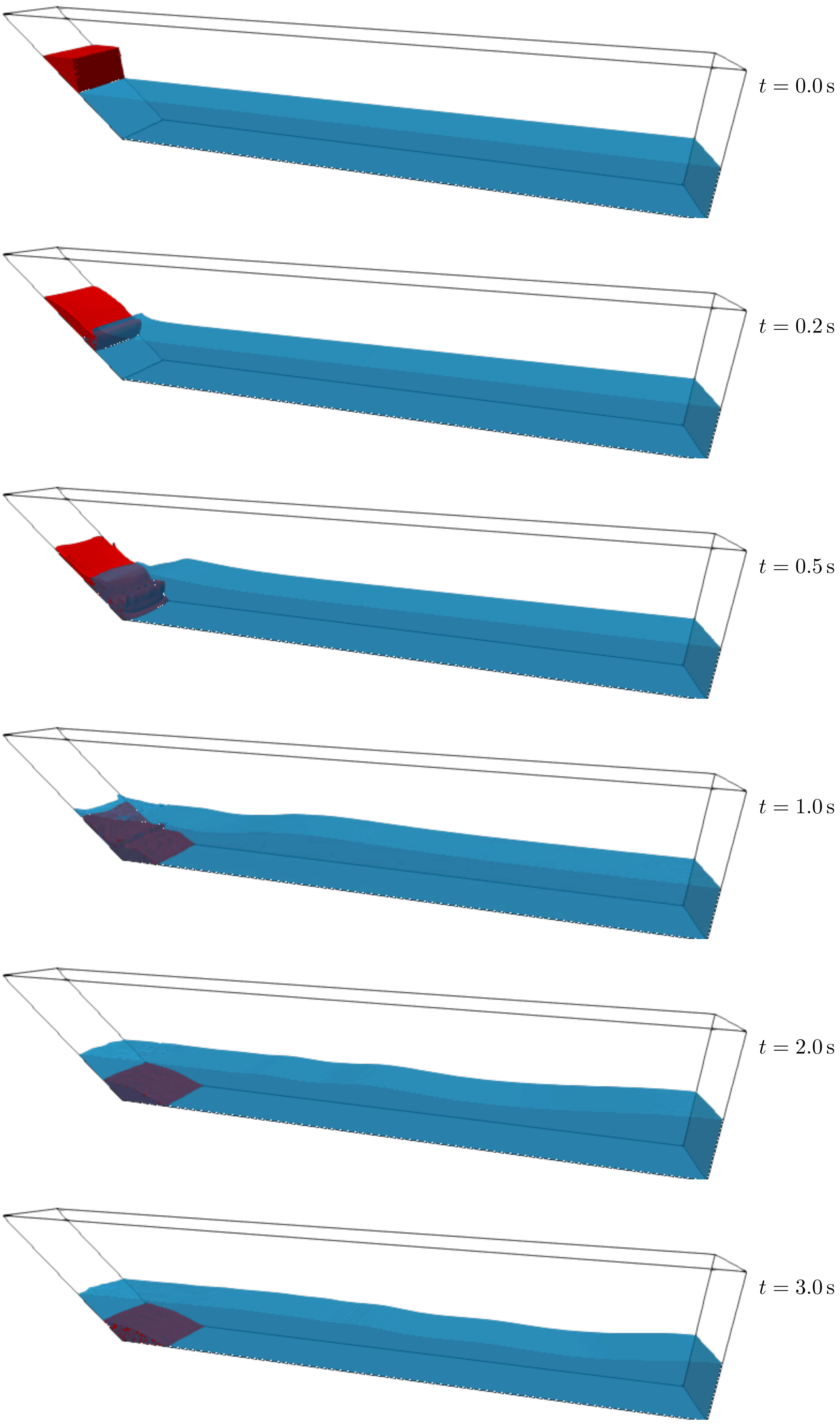

Figure 35: Perspective view of a 3D simulation of the laboratory experiment.

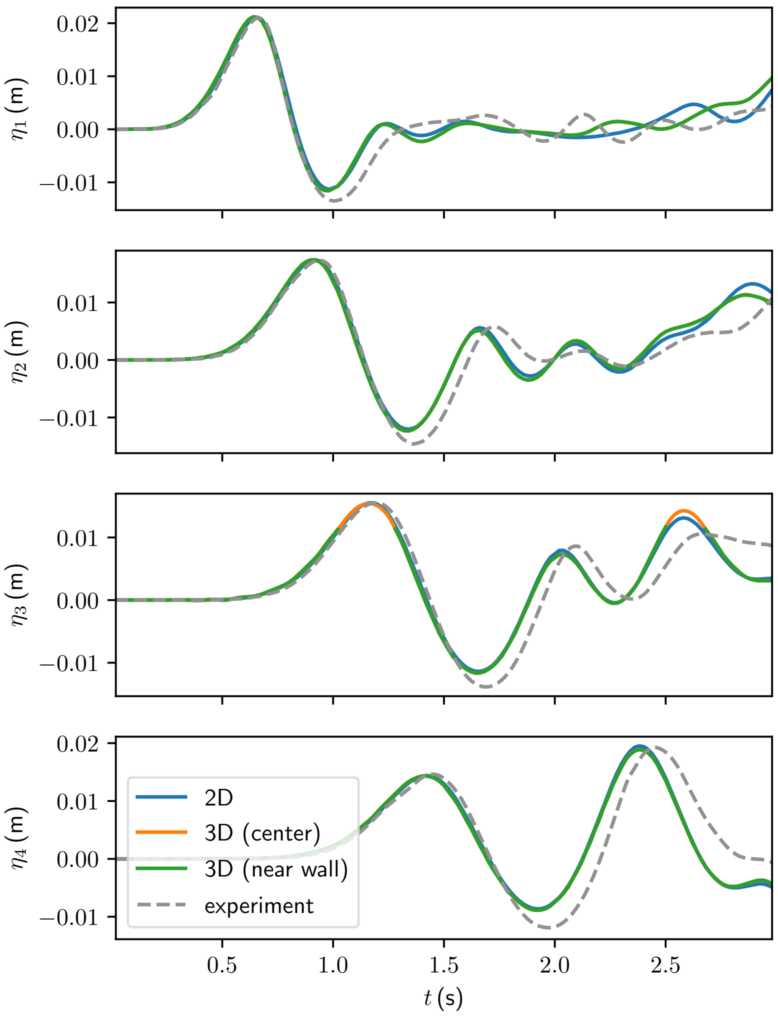

Figure 36: Wave gauges, positioned in the middle and close to the side wall for the three-dimensional laboratory experiment simulation in comparison to the two-dimensional simulation of the same experiment.

7 Sensitivity analysis of Lake Askja landslide

The three-dimensional simulations of the Lake Askja landslide tsunami are numerically expensive and a full sensitivity analysis can hardly be undertaken. However, the question on the sensitivity on the friction coefficient was pressing and we conducted an additional simulation with a reduced friction coefficient (from $\mu_\mathrm{s} = 0.17$, $\mu_\mathrm{d} = 0.37$ to $\mu_\mathrm{s} = 0.14$, $\mu_\mathrm{d} = 0.34$). The reduced friction coefficient was chosen because the friction coefficient that was derived from the slide deposition underestimated the inundation on the southern shore. The simulation was ran until $t = 240\,\mathrm{s}$ as most of the inundation happens within this period.

The slide velocity increases with the reduced friction angle (see Fig. 38) but the runout is not substantially affected because the slide is stopped by an elevation in the lake bathymetry (see Fig. 38). The wave height increases similarly with the slide velocity (see Fig. 39) and the same is the case for the inundation (see Fig. 37). The formerly underestimated inundation on the southern shore is partially fitted better but also overestimated in some regions. The already overestimated inundation on the northern shore is further overestimated, the underestimated inundation in the far field on the western shore increases with a lower friction angle and fits the field data better. Overall, the fitness is better with the static friction coefficient of $\mu_\mathrm{s} = 0.17$ (dynamic friction coefficient $\mu_\mathrm{d} = \mu_\mathrm{s} + 0.2 = 0.37$), corresponding to a friction angle of $10°$, see Tab. 20.

An important lesson from this comparison is that the southern shore and the northern shore cannot be fitted at the same time with a homogenous initialisation of the landslide. The simulated tsunami is stronger on the northern side than on the southern side, in comparison with the field observations. The same is the case for the slide and its runout is overestimated on the northern side for both scenarios (see Fig. 38). The conclusion can only be that the slide was in reality oriented further towards the south and this indicates that the failure was initialized on the southern side and that the bulk of the collapsing slope was flowing stronger in the direction of that initial failure. This was not considered in the simulation and the whole slide release was treated as a homogenous bulk.

Table 20: Errors in the inundation for two different friction angles.

μ_s	μ_d	L2 error	mean error	maximum error
0.17	0.37	10.6 m	8.3 m	32.2 m
0.14	0.34	12.5 m	10.0 m	42.3 m

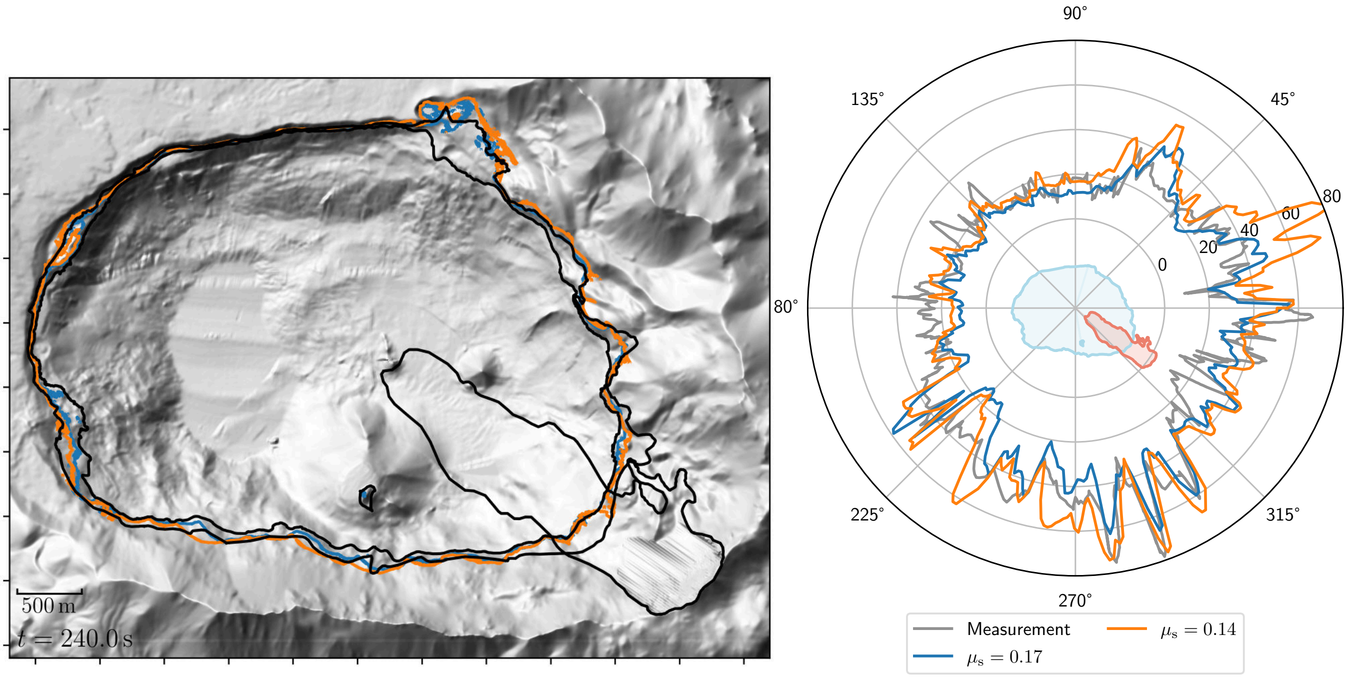

Figure 37: Comparison of the inundation at $t = 240\,\mathrm{s}$ between $\mu_\mathrm{s} = 0.17$, $\mu_\mathrm{d} = 0.37$ and $\mu_\mathrm{s} = 0.14$, $\mu_\mathrm{d} = 0.34$.

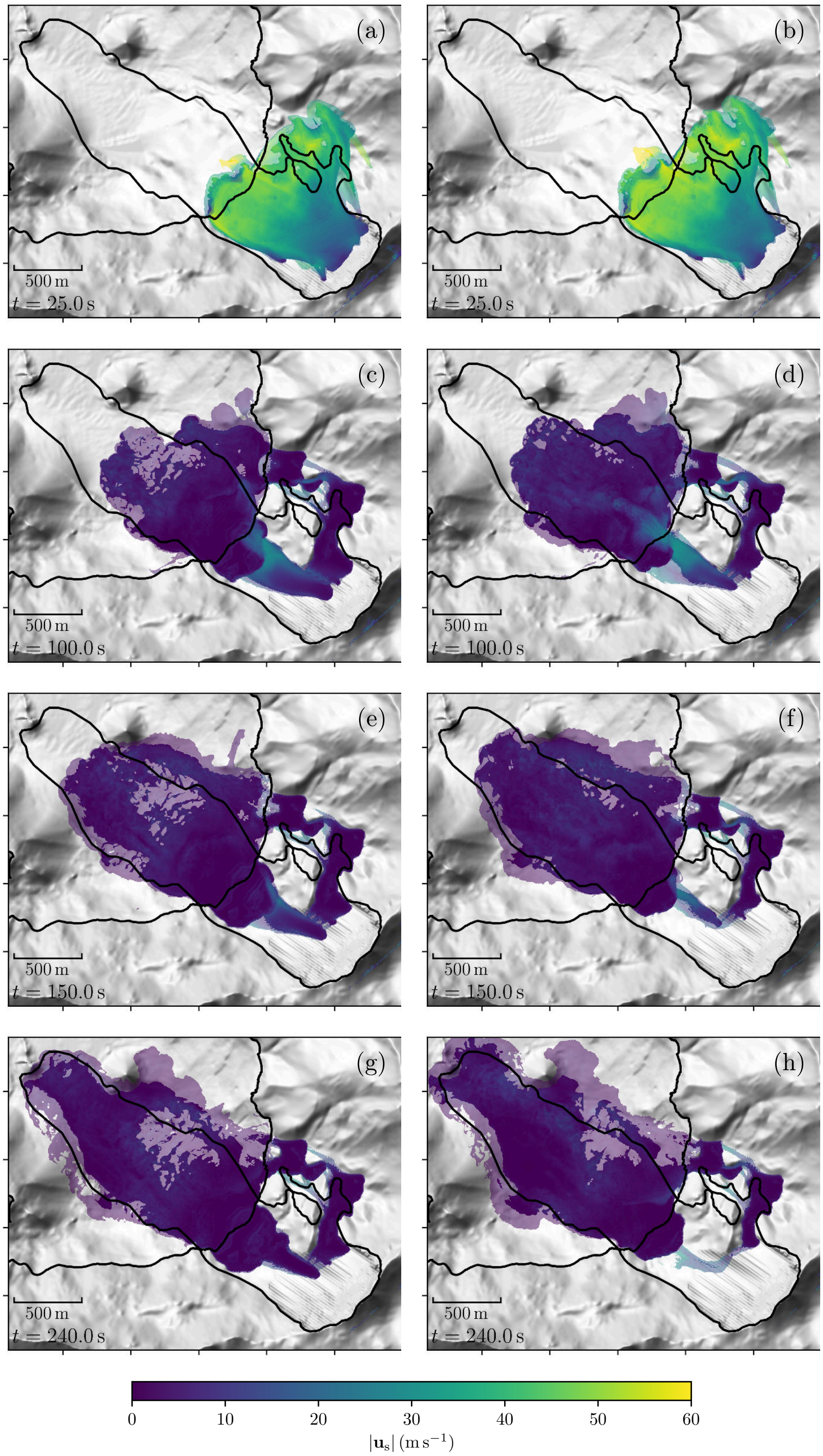

Figure 38: Comparison of the slide between a friction coefficient of $\mu_s = 0.17$, $\mu_d = 0.37$ (left: a,c,e,g) and $\mu_s = 0.14$, $\mu_d = 0.34$ (right: b,d,f,h).

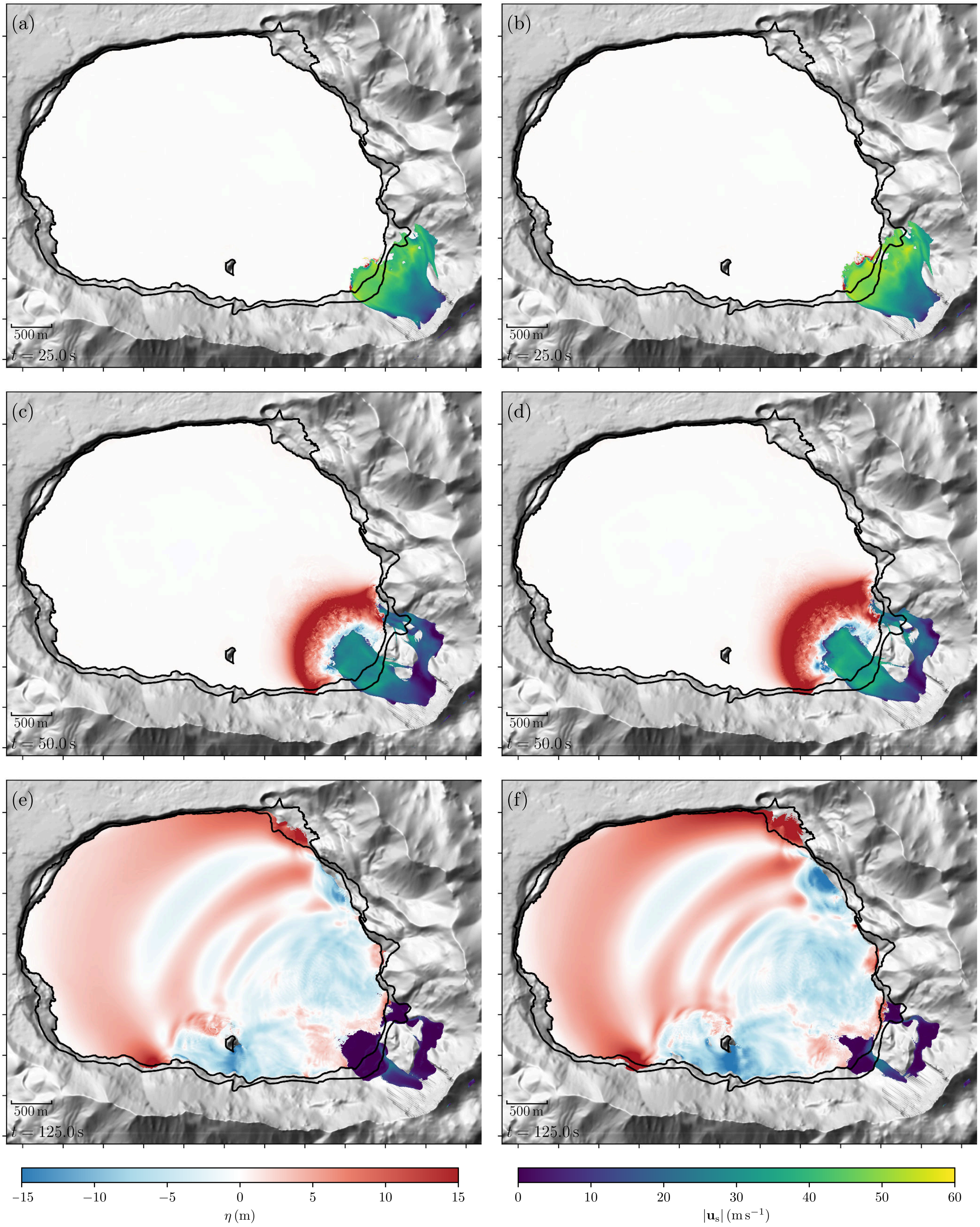

Figure 39: Comparison of the wave between a friction coefficient of $\mu_\mathrm{s} = 0.17$, $\mu_\mathrm{d} = 0.37$ (left: a,c,e) and $\mu_\mathrm{s} = 0.14$, $\mu_\mathrm{d} = 0.34$ (right: b,d,f).

8 Centre of mass of slide and water phase in the Lake Askja case

The centre of mass $\mathbf{c}_i$ within a control volume Ω of a phases ϕ_i can be calculated as

$$\mathbf{c}_i = \frac{1}{m_i} \int_\Omega \rho_i \, \phi_i \, \mathbf{x} \, \mathrm{d}\mathbf{x}, \tag{48}$$

where m_i is the total mass of phase ϕ_i calculated as

$$m_i = \int_\Omega \rho_i \, \phi_i \, \mathrm{d}\,\mathbf{x}. \tag{49}$$

The control volume Ω spans over the whole simulation domain to get the overall centre of mass of the phase. The velocity of the centre of mass can be similarly calculated as

$$\mathbf{u}_{\mathrm{c},i} = \frac{1}{m_i} \int_\Omega \rho_i \, \phi_i \, \mathbf{u}_i \, \mathrm{d}\mathbf{x}. \tag{50}$$

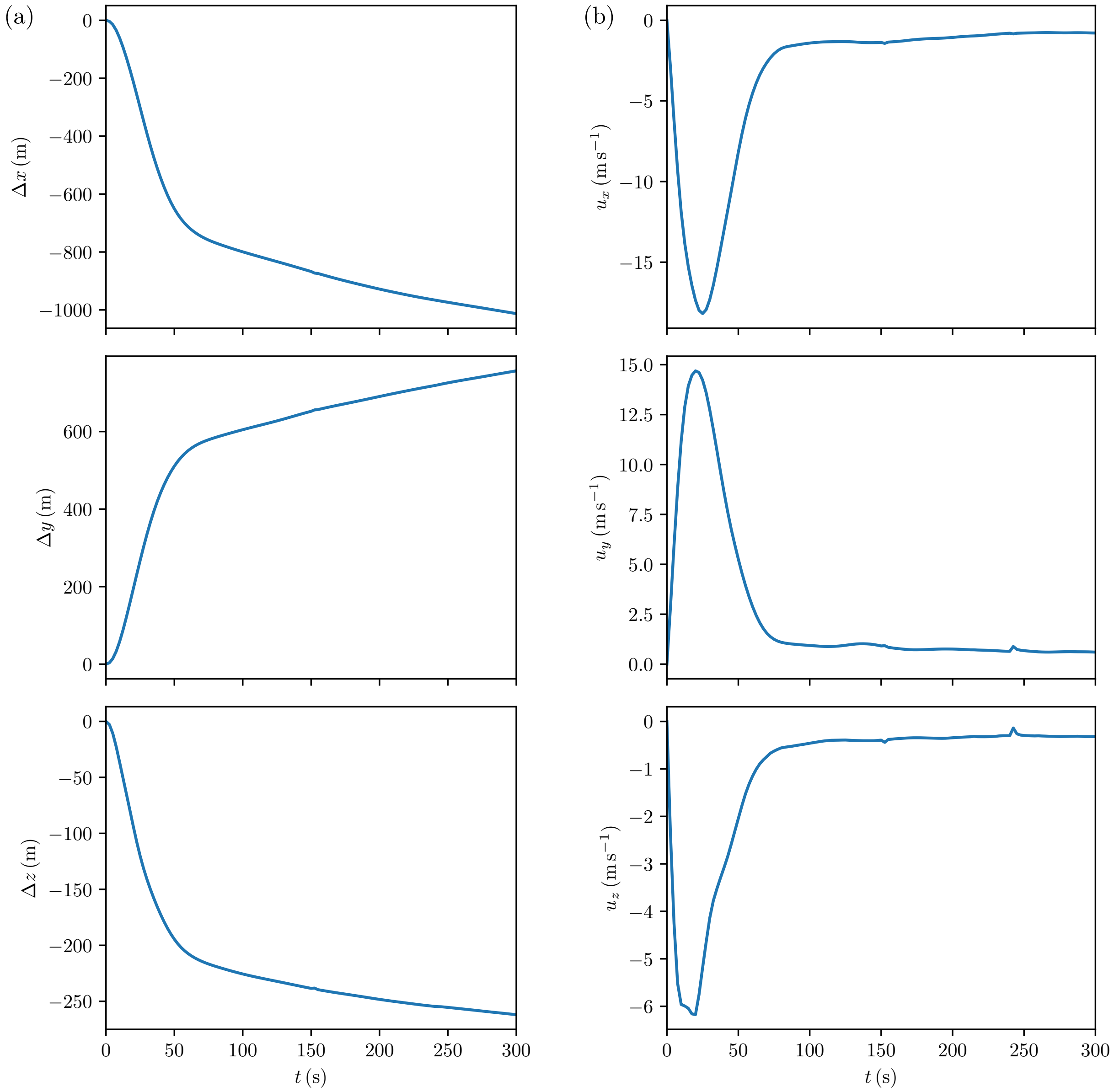

Figure 40: Centre of mass displacement (a) and velocity (b) of the slide phase.

The displacement of the centre of mass in relation to the centre of mass at $t = 0\,\mathrm{s}$ is shown in Fig. 40a for the slide (i.e. the granular phase $\phi_i = \phi_\mathrm{g}$) alongside its velocity in Fig. 40b. The initial acceleration between $t = 0\,\mathrm{s}$ and $t = 25\,\mathrm{s}$, as well as the rapid deceleration between $t = 25\,\mathrm{s}$ and $t = 50\,\mathrm{s}$ can be observed well. The velocity of the centre of mass in the later parts of this event is low and only parts of the slide keep moving slowly.

The same processing is shown in Fig. 41 for the water body (i.e. the water phase $\phi_i = \phi_\mathrm{w} = \phi_\mathrm{c}\,\alpha_\mathrm{w}$). The elevation of the centre of mass of the water phase up to 0.6 m is shown well in this figure. We expect that the elevation would be reduced if the simulation was ran longer, as air enclosed in the slide would eventually rise to the top (water would sink respectively). Further, the agitated surface of the lake would calm down. The data shown here fits the observations of Gylfadóttir et al. [4] of a rising water level of $0.65 \pm 0.2\,\mathrm{m}$ well.

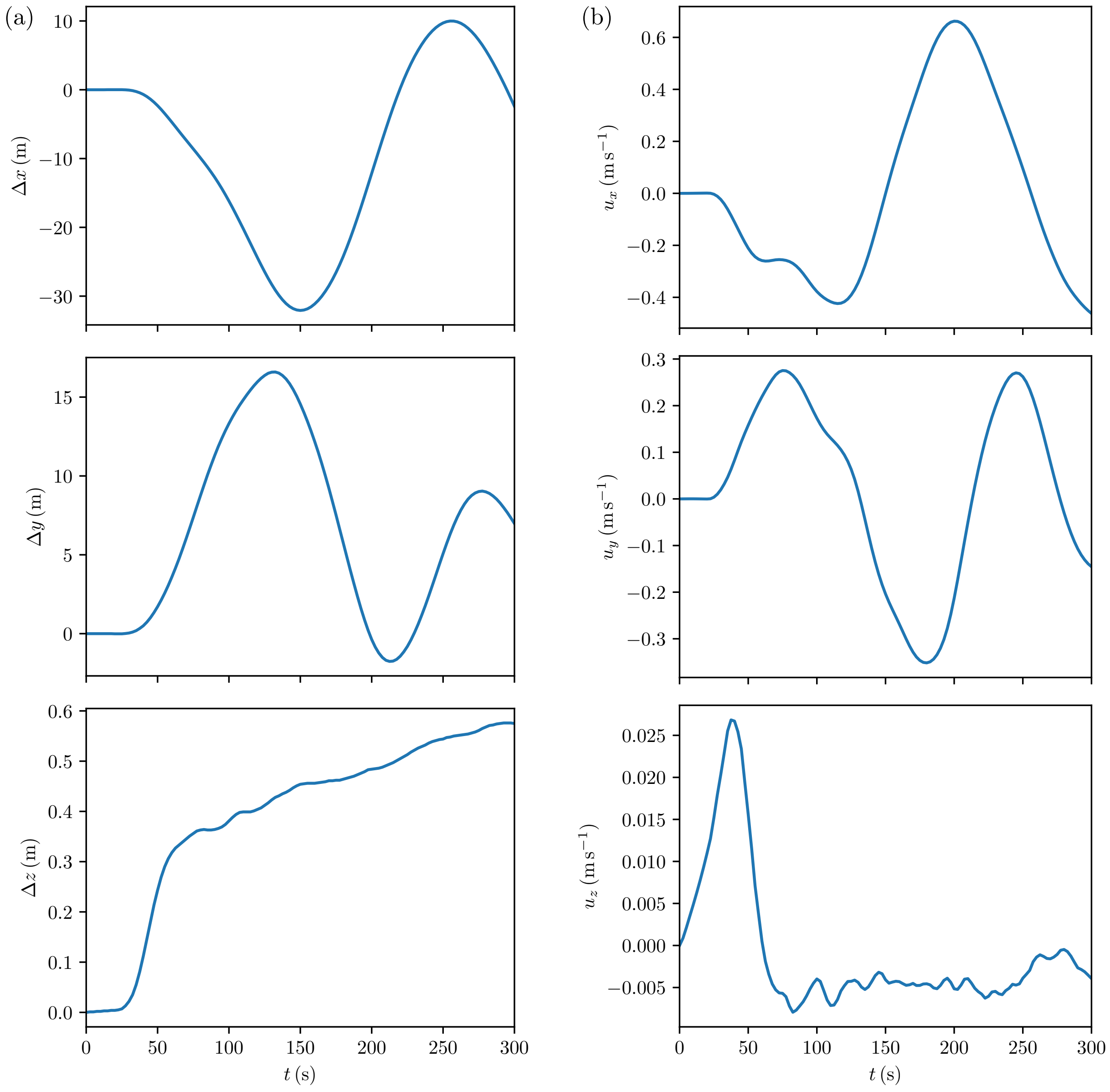

Figure 41: Centre of mass displacement (a) and velocity (b) of the water phase.

References

[1] M. Rauter, "The compressible granular collapse in a fluid as a continuum: validity of a Navier-Stokes model with $\mu(J)$,$\phi(J)$-rheology," *Journal of Fluid Mechanics*, vol. 915, 2021.

[2] M. Rauter, A. Kofler, A. Huber, and W. Fellin, "faSavageHutterFOAM 1.0: depth-integrated simulation of dense snow avalanches on natural terrain with OpenFOAM," *Geoscientific Model Development*, vol. 11, no. 7, pp. 2923–2939, 2018.

[3] F. Juretić, *cfMesh user guide*. Creative Fields, Zagreb, 2015.

[4] S. S. Gylfadóttir, J. Kim, J. K. Helgason, S. Brynjólfsson, Á. Höskuldsson, T. Jóhannesson, C. B. Harbitz, and F. Løvholt, "The 2014 Lake Askja rockslide-induced tsunami: Optimization of numerical tsunami model using observed data," *Journal of Geophysical Research: Oceans*, vol. 122, no. 5, pp. 4110–4122, 2017.

[5] M. Rauter, L. Hoße, R. Mulligan, A. Take, and F. Løvholt, "Numerical simulation of impulse wave generation by idealized landslides with OpenFOAM," *Coastal Engineering*, vol. 165, p. 103815, 2021.

[6] J. Roenby, B. E. Larsen, H. Bredmose, and H. Jasak, "A new volume-of-fluid method in OpenFOAM," in *VII International Conference on Computational Methods in Marine Engineering. Nantes: International Center for Numerical Methods in Engineering*, 2017.

[7] S. Viroulet, A. Sauret, O. Kimmoun, and C. Kharif, "Granular collapse into water: toward tsunami landslides," *Journal of Visualization*, vol. 16, no. 3, pp. 189–191, 2013.

[8] H. K. Versteeg and W. Malalasekera, *An introduction to computational fluid dynamics: the finite volume method*. Pearson education, 2007.

[9] F. Chen, V. Heller, and R. Briganti, "Numerical modelling of tsunamis generated by iceberg calving validated with large-scale laboratory experiments," *Advances in Water Resources*, vol. 142, p. 103647, 2020.
