# Supplementary material for: Granular porous landslide tsunami modelling – the 2014 Lake Askja flank collapse
Source: Nat Commun. 2022 Feb 3;13:678. doi: 10.1038/s41467-022-28296-7 (PMC8813955; doi:10.1038/s41467-022-28296-7)
Supplement: Supplementary file 3 — Description of Additional Supplementary Files [file 41467_2022_28296_MOESM3_ESM.pdf]

Title: Supplementary Movie 1:
Description: The simulated Askja landslide. Animated version of Fig. 5. The dense core is represented by the $\phi g = 0.25$ iso-surface and the surrounding dilute particle cloud by the $\phi g = 0.01$ iso-surface. The colour shows the particle velocity and the dilute cloud is shown transparent. The black lines show the lake shoreline and the documented avalanche path for reference. For the colour map, see Fig. 5 in the article.

Title: Supplementary Movie 2:
Description: The simulated Askja landslide (dense core). Same as movie 01 but without the powder cloud, giving an unhindered view on the dense core. The dense core is represented by the $\phi g = 0.25$ iso-surface. The colour shows the particle velocity. The black lines show the lake shoreline and the documented avalanche path for reference. For the colour map, see Fig. 5 in the article.

Title: Supplementary Movie 3:
Description: The simulated Askja landslide tsunami. Animated version of Fig. 6 in the article, showing the dense core of the landslide and the tsunami in the lake. For the colour map, see Fig. 6 in the article.

Title: Supplementary Movie 4:
Description: The simulated Askja landslide tsunami, perspective view. The Lake Askja landslide including the fluidized powder cloud and the generated lake tsunami. The fluidized cloud is shown slightly transparent. For the colour map, see Fig. 6 in the article.

Title: Supplementary Movie 5:
Description: Vertical slice through the Askja landslide tsunami. Animated version of Fig. 7 in the article, showing a cross section through the landslide and the tsunamigenic region. The colour represents the local phase fractions. The free water surface and the mesh boundary are highlighted as a black line. For the colour map, see Fig. 7 in the article
